# Influence of the Origin, Feeding Status, and *Trypanosoma cruzi* Infection in the Microbial Composition of the Digestive Tract of *Triatoma pallidipennis*

**DOI:** 10.3390/biology14080984

**Published:** 2025-08-02

**Authors:** Everardo Gutiérrez-Millán, Alba N. Lecona-Valera, Mario H. Rodriguez, Ana E. Gutiérrez-Cabrera

**Affiliations:** 1 Centro de Investigación Sobre Enfermedades Infecciosas, Instituto Nacional de Salud Pública, Avenida Universidad 655, Col. Santa María Ahuacatitlán, Cerrada Los Pinos y Caminera, Cuernavaca 62100, Morelos, Mexico; ever.gmillan@gmail.com (E.G.-M.); alba.lecona@insp.mx (A.N.L.-V.); 2 SECIHTI Adscribed to Centro de Investigación Sobre Enfermedades Infecciosas, Instituto Nacional de Salud Pública, Cuernavaca 62100, Morelos, Mexico

**Keywords:** *Triatoma pallidipennis*, *Trypanosoma cruzi*, bacterial community, wild insects, insectary insects, core microbiota

## Abstract

*Triatoma pallidipennis*, the main vector of Chagas disease in central Mexico, hosts a diverse and complex gut bacterial community shaped by environmental and physiological factors. To gain insight into these microbes’ dynamics, we characterised the gut bacterial communities of wild and insectary insects under different feeding and *Trypanosoma cruzi* infection conditions, using 16S rRNA gene sequencing. We identified 91 bacterial genera across 8 phyla, with Proteobacteria dominating most samples. Wild insects showed greater bacterial diversity, led by *Acinetobacter* and *Pseudomonas,* while insectary insects exhibited lower diversity and were dominated by *Arsenophonus*. The origin of the insects, whether they were reared in the insectary (laboratory) or collected from wild populations, was the principal factor structuring the gut microbiota, followed by feeding and *T. cruzi* infection. A stable core microbiota of 12 bacterial genera was present across all conditions, suggesting key functional roles in host physiology. Co-occurrence and functional enrichment analyses revealed that feeding and infection induced condition-specific microbial interactions and metabolic pathways. Our findings highlight the ecological plasticity of the triatomine gut microbiota and its potential role in modulating vector competence, providing a foundation for future microbiota-based control strategies.

## Simple Summary

*Triatoma pallidipennis* is an insect vector that lives in central Mexico and transmits Chagas disease. This study investigated how the bacteria living in the gut of these insects change depending on where they live, if they have fed on blood, or if they carry the parasite that causes the disease. We studied wild and raised-in-the-lab individuals. Wild insects had more sorts of bacteria, while lab-raised ones had fewer. However, all of them shared 12 bacterial types, which might be important for the insect’s biology. The results showed that feeding and infection influence how these bacteria interact. This research helps to understand how bacteria participate in the physiology of these insects and how they might influence Chagas disease spread.

## 1. Introduction

The digestive tracts of insects are among the most complex and diverse microbial ecosystems [1,2]. Their composition and structure may be influenced by their functional status and the environment the insects are exposed to [3], which in turn modulate the acquisition, expansion, or extinction of microbial groups [4]. In these systems microbial diversity varies depending on the morphology of the organ [5], its physicochemical properties [6,7], and diet [8]. In hematophagous insects the digestive track microbiome contributes to nutrition [9], protection against parasites, and competence to transmit pathogens [10,11]. In insects that feed exclusively on blood, microbiomes are dominated by specialised, evolutionary ancient symbiotic bacteria with biosynthetic capabilities that complement dietary limitations [12]. In some insects these bacteria can be replaced by others that are better able to colonise the host [13,14].

There are 152 triatomine species, distributed mainly on the American continent [15]. All are hematophagous and considered vectors of the protozoan *Trypanosoma cruzi*, the causal agent of Chagas disease, which puts at least 70 million people at risk in Latin America [16]. The triatomine digestive tract is divided into three regions—anterior/stomach, middle/intestine, and posterior/rectum—where *T. cruzi* parasites are exposed to the vector’s immune system and digestive enzymes and are in close relation with the bacteria present in each region [17,18]. Factors such as habitat [19], the presence of blood and *T. cruzi*, and nutritional status, in addition to bacterial population interactions, appear to be important modulators of the microbiome. In this study, we investigated the presence and abundance of bacterial genera in the digestive tract of wild and colonised *Triatoma pallidipennis* under fasting and blood-feeding conditions, as well as the possible effect of *T. cruzi* infection. In central Mexico, *T. pallidipennis* is an important vector of Chagas disease [20], which commonly inhabits peridomestic and urbanised areas [21]. Wild specimens were collected from their typical habitats, while insectary-reared individuals were kept under controlled laboratory conditions. This comparison allowed us to evaluate the impact of habitat complexity and environmental exposure on gut microbiota composition. Our analysis allowed the identification of bacteria genera common to all conditions, which likely constitute permanent microbiota as they were consistently detected regardless of insect origin (wild or insectary), feeding status, or *T. cruzi* infection. Bacteria present only in wild insects seem to represent transient colonisers, while other genera seem to be influenced by blood feeding. The positive and negative association observed between bacteria and *T. cruzi* may reflect the direct effects of the parasite on specific genera.

## 2. Materials and Methods

### 2.1. Conditions of T. pallidipennis from Insectary and Wild Environments

Specimens of *T. pallidipennis* used in this study belonged to the second laboratory generation, derived from specimens originally collected in peridomestic environments. They were reared in cages at 28 ± 2 °C and 65 ± 5% relative humidity in the insectary of the Centre for Research in Infectious Diseases at the National Institute of Public Health in Mexico. Insects were fed rabbit blood using artificial feeders. Wild triatomes were collected from multiple peridomestic sites across the state of Morelos, Mexico, between May and September 2019, and were processed immediately after their collection.

Specimens were distributed in groups of six individuals according to their origin, feeding status, and infection with *T. cruzi* as follows:

(A) Insectary, fasting, not infected;
(B) Insectary, blood fed, not infected;
(C) Insectary, fasting, infected with *T. cruzi*;
(D) Insectary, blood fed, infected with *T. cruzi*;
(E) Wild, unfed not infected;
(F) Wild, blood fed, not infected;
(G) Wild, unfed, infected with *T. cruzi*;
(H) Wild, blood fed, infected with *T. cruzi*.

To form the insectary groups, recently moulted 5th instar nymphs were selected and subjected to a fasting period for 30 ± 5 days. At the end of this period, Group A was dissected, while Group B was blood fed and dissected eight days later. For the groups infected with *T. cruzi*, 4th instar nymphs were fed with *T. cruzi*-infected blood. After moulting to 5th instar, they were subjected to fasting for 30 ± 5 days. Group C was dissected after this period. Group D was fed with uninfected blood and dissected eight days later. Wild 5th instar nymph specimens were grouped according to their condition at the time of capture into empty abdomens not infected with *T. cruzi* (Group E), full abdomens not infected with *T. cruzi* (Group F), empty abdomens infected with *T. cruzi* (Group G), and full abdomens infected with *T. cruzi* (Group H). The presence of *T. cruzi* was investigated by light microscopy (Primo Star, ZEISS 40X) in all groups. Microscopy-negative specimens were confirmed by amplification of the mini-exon SL gene using Polymerase Chain Reaction [22]. All individuals were dissected to obtain their digestive tracts, which were segmented into three regions (anterior/stomach, middle/intestine, and posterior/rectum) and individually processed, completing a total of 109 samples.

### 2.2. Detection of T. cruzi by Amplification of the SL Mini-Exon Gene

The amplification of the non-transcribed spacer of the intergenic region of the mini-exon gene [22] was carried out using a multiplex PCR assay with a pool of oligonucleotides: TcI:5′GTGTCCGCCACCTCCTTCGGGCC (specific to TcII to TcVI groups), TcII: 5′GTGTCCGCCACCTCCTTCGGGCC (specific to the TcI groups), and Tc: 5′CCCCCCTCCCAGGC CACACTG (common oligonucleotide of the TcI to TcVI strains). The thermal profile was 94 °C/ 1 min; 27 cycles of 94 °C/30 s, 55 °C/30 s, 72 °C/30 s; and 72 °C/10 min. Amplification products were analysed in 2% agarose gel [22].

### 2.3. Blood Feeding and T. cruzi Infection

To feed and infect insects we used parafilm membranes on artificial feeders at 34 °C. They were provided with heat-inactivated New Zealand rabbit blood added with 30 U/mL of heparin (Sigma, St. Louis, MO, USA) [23]. Blood was centrifuged at 2500 rpm for 10 min [24]. The plasma was subjected to 55 °C for 30 min [25]. The globular package was washed with phosphate-buffered saline (PBS, 137 mM NaCl, 2.7 mM KCl, 10 mM Na_2_HPO_4_, 1.8 mM KH_2_PO_4_) and centrifuged at 2500 rpm for 10 min at room temperature. The plasma and globular package were reconstituted, and *T. cruzi* epimastigotes (4 × 10^6^ parasites/mL; ITPHI/MX/19/CEPA-ANTONIO-19 donated by de Fuentes-Vicente, José Antonio—UNICACH) were added to the mixture [23]. Parasites were previously cultured in Liver Infusion Tryptose (LIT) medium (Sigma-Aldrich, USA), prepared according to the method described by Chiari and Camargo (1984) [26], and composed of 68.4 mM NaCl, 5.3 mM KCl, 29.5 mM Na_2_HPO_4_, 11.1 mM glucose, 0.5% liver infusion, 0.5% tryptose, 0.0025% hemin, 10% foetal bovine serum (FBS), and 1X Penicillin–Streptomycin–Neomycin (PSN) antibiotic mixture (Thermo Fisher Scientific, Waltham, MA, USA). Parasites were harvested during an exponential growth phase, centrifuged at 2500 rpm for 3 min at room temperature, and counted in Neubauer chambers [23].

### 2.4. Preparation of Samples from the Digestive Tract and DNA Extraction

The digestive tracts of individual insects were processed separately under sterile conditions. Prior to dissection, insects were treated with benzalkonium chloride 0.1% (benzalkonium chloride Altamirano, CDMX, Mexico) and washed with ultrapure Milli-Q Type I water (resistivity 18.2 MΩ·cm, produced with a PURELAB Classic system, ELGA LabWater, Woodridge, IL, USA). To avoid contamination the abdominal connective tissue was cut and the digestive tract was exposed, removed, and placed on a plate with sterile PBS. Each segment of the digestive tract was separated into a 2 mL microtube (Eppendorf, Enfield, CT, USA) with sterile 1.4 mm ceramic beads (Omni International, Kennesaw, GA, USA) to produce tissue lysis. The samples were processed to obtain total DNA, using the DNeasy Blood & Tissue Kit (QIAGEN, Hilden, Germany) protocol. The quality and quantity of DNA were assessed by electrophoresis in 0.8% agarose gels and quantified with a QIAxpert UV/VIS spectrophotometer (QIAGEN, Hilden, Germany).

### 2.5. Amplification of the Bacterial 16S rRNA Gene

The first amplification targeted a 476 bp fragment encompassing both the V3 and V4 hypervariable regions of the bacterial 16S rRNA gene, using primers 341F (5′-CCTACGGGNGGCWGCAG-3′) and 805R (5′-GACTACHVGGGTATCTAATCC-3′) which provide sufficient variability to enable differentiation at the genus level [27]. A second PCR was performed with oligonucleotides 341F and 805R with adapters of the MiSeq massive sequencing platform (2 × 300 bp) of Illumina according to the protocol “16S Metagenomic Sequencing Library Preparation” [28]. Each library was encoded with identifying sequences using the 96-sample Nextera XT Index Kit [29] system. PCR with adapters, quantification, and purification of PCR products (AMPureX, Indianapolis, IN, USA), as well as amplicon processing, were performed by the sequencing services (MiSeq platform) [30] of the National Institute of Genomic Medicine, CDMX, Mexico.

### 2.6. Filtering 16S Sequenced Reads

The data obtained from sequencing were subjected to a two-stage filtering process. In the first, low-quality reads were removed using the Trimmomatic v.38 tool [31]. In the second, the oligonucleotides associated with the 16S amplicons were eliminated, using the cutadapt v1.8 tool [32]. Finally, the default parameters were used for paired sequences.

### 2.7. Construction of Amplicon Sequence Variants (ASVs) of 16S Sequences

Amplicon Sequence Variants (ASVs) represent unique sequences derived from amplicon sequencing data [27]. For the construction of ASVs, the filtered reads were used as input in the R DADA2 v1.24 package [33] (https://benjjneb.github.io/dada2/, accessed on 16 February 2025). In this process, sequences with lengths of less than 400 bases were excluded. To do this the error rate for each sequence was calculated by default and redundancy was eliminated. The paired sequences were fused, and possible chimaeras were ruled out. Thus, ASVs are an accurate and detailed way representing microbial diversity in amplicon sequencing data, providing higher taxonomic resolution. Taxonomic classification was performed using the Silva v138.1 database (https://zenodo.org/records/4587955#.YtGRqexBz0o, accessed on 16 February 2025), assigning most ASVs to the genus level. A phylogenetic tree of the classified data was then constructed using the R package phangorn v2.11.1 [34] to estimate the phylogenetic distances among the analysed samples. The tree was initially built with the neighbour-joining method and subsequently refined using the Maximum Likelihood (ML) approach [35], with 1000 bootstrap replicates. Branches with bootstrap support values of 70% or higher were considered well supported.

### 2.8. Analysis of the Composition, Structure, Abundance and Diversity of the Microbiota

We used the phyloseq package (v1.4) (https://github.com/joey711/phyloseq, accessed on 16 February 2025) [36] in R (v4.2.1) to organise the microbial data into a single object that included the ASVs, taxonomy, metadata (such as the sample origin, gut section, and experimental condition), and the phylogenetic tree. To reduce noise from low-frequency features, we filtered out ASVs that appeared in fewer than three samples within any group or that showed a relative abundance below 0.01%. After this step we converted the data to relative abundances and applied a log_2_ transformation for downstream comparisons. To examine how the microbiota varied among samples, we used Principal Coordinates Analysis (PCoA) based on weighted UniFrac distances [37]. This approach helped us to visualise patterns by combining phylogenetic relatedness with abundance. We then tested for statistical differences across groups using PERMANOVA (adonis2 function in R package vegan, v2.6.4) [38,39], with significance considered at *p*-value < 0.05. Shannon’s index (H′) was used to measure alpha diversity [27], calculated through the diversity function in vegan. Before comparing diversity values between groups, we assessed data normality using the Shapiro–Wilk test (shapiro. test). In cases where assumptions of normality were not met, we used the Wilcoxon rank-sum test (wilcox. test) instead [39]. The same method was applied to test differences in relative abundances between experimental conditions.

### 2.9. Differential Abundance and Microbiota Correlation Analysis

To investigate variations in bacterial composition under the conditions studied (origin, feeding, and infection with *T. cruzi*), differential abundance analyses were conducted using the Wald tests implemented in the DESeq2 v1.36 package [40]. Significant differences between conditions were identified, with *p*-values adjusted using the Benjamini–Hochberg false discovery rate (FDR) method to achieve a threshold of FDR ≤ 0.05 [41]. To identify meaningful changes in bacterial abundance at the genus level, we applied a log_2_-fold-change cutoff of ≥1.5 or ≤−1.5 [42,43,44]. Additionally, microbiota correlation analyses were performed to explore statistical relationships among microbial taxa, providing insights into the structures and dynamics of the bacterial communities.

### 2.10. Microbial Co-Occurrence Network Analysis

To assess microbial interactions, genus-level co-occurrence networks were constructed based on microbiota data from insectary and wild insects. We used the R package phylosmith (v1.0.7) [45], starting with a phyloseq object containing taxonomic and abundance data. First, the phyloseq object was filtered to generate data subsets corresponding to each condition. ASVs were then aggregated at the genus level, creating a new *phyloseq* object, which was subsequently used for co-occurrence network construction. The co-occurrence network was generated using the co_occurrence_network () function of the phylosmith package, with Spearman’s correlation as the metric for evaluating significant associations between genera. A significance threshold of *p*-values ≤ 0.01 and a correlation coefficient (rho) ≥ 0.8 were applied, focusing on strong correlations. The resulting networks provided insights into both positive and negative correlations between genera based on their abundance under each condition, enabling the identification of potential ecological interactions within the microbiota. Similarly, “modularity” describes the presence of distinct clusters (modules) of bacterial genera that are more strongly connected to each other than to the rest of the network. High modularity indicates structured sub-communities, while a loss of modularity reflects a more uniform and less specialised network.

### 2.11. Inferences and Enrichment of Functional Profiles

To identify over-represented biological functions, we performed a functional enrichment analysis based on the Kyoto Encyclopaedia of Genes and Genomes (KEGG) orthologs (KOs) abundance profiles predicted with PICRUSt2 (v2.6.0) [46]. This tool infers gene content from 16S rRNA sequences by placing them in a reference phylogenetic tree and assigning functions based on related reference genomes. Predictions with NSTI (Nearest Sequenced Taxon Index) > 0.15 were excluded to ensure reliability. KO abundance matrices were normalised (relative abundances), log_2_ transformed (pseudo count + 1), and mean KO abundances were calculated. The top 10%—those above the 90th percentile—were used for Over-Representation Analysis (ORA) based on the hypergeometric distribution via the enricher () function in the clusterProfiler R package [47], using KEGG annotations (ko2kegg_category.tsv and ko2pathway.tsv from https://www.kegg.jp/kegg, accessed on 16 April 2025). Only pathways with an adjusted *p*-value ≤ 0.01—Benjamini–Hochberg correction—were retained. To assess condition-specific activity, we calculated Z-score per pathway using the normalised KO data. Positive Z-scores indicate upregulation, negative values indicated downregulation. Only pathways with an adjusted *p*-value (FDR) < 0.01 were considered for interpretation. Additionally, we identified enriched functional pathways from differentially abundant KOs using DESeq2, comparing fasting vs. feeding and infection condition with/without *T. cruzi* in both insectary and wild groups. KOs with |log_2_FC| ≥ 2 and FDR < 0.01 were analysed using enricher () to identify overrepresented pathways (*p*-value adjusted < 0.05 using the Benjamini–Hochberg correction).

## 3. Results

### 3.1. Summary of Sequenced Samples

One hundred and nine samples met the conditions of the concentration and quality of genomic material. Of these, 56 were from wild specimens and 53 from insectary specimens. The numbers of samples from all groups are presented in Supplementary Table S1. Reads from digestive tract samples accounted for 94% (6,626,583) of the total reads (7,022,734). The final selected reads included 333 ASVs that were present in at least three samples of the same group and had a relative abundance equal to or greater than 0.01% of the total analysed reads.

### 3.2. General Overview of the Gut Microbiota

**Figure 1:**
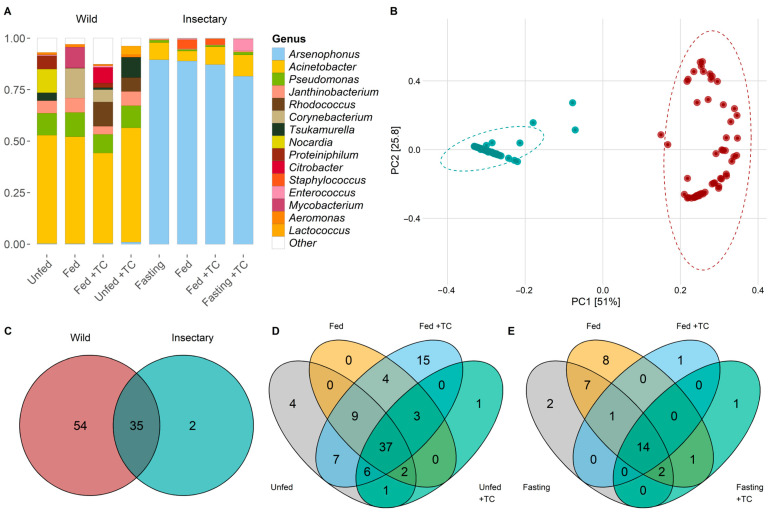
Structure and composition of gut microbiota of wild and insectary *T. pallidipennis*. (**A**) Barplot of the fifteen most common bacterial species according to log_2_-transformed relative abundances. Colours identify different genera; white denotes low-abundance taxa gathered as “Other”. Wild samples show higher diversity, with *Acinetobacter* and *Pseudomonas* predominating, while insectary insects are dominated by *Arsenophonus*. (**B**) PCoA based on weighted UniFrac distances shows clear separation between wild (red) and insectary (blue) samples, confirming origin as the main driver structuring the microbiota (PC1: 51%, PC2: 25.8%). (**C**) Venn diagrams comparing genera between wild (red) and insectary (blue) samples. Shared genera between wild and insectary insects are depicted in the overlapping section of the Venn diagram, indicating a conserved microbiota across origins. Venn diagrams depict species distribution across conditions in wild (**D**) and insectary (**E**) insects. Colours represent each state: grey (unfed), orange (fed), light blue (fed + *T. cruzi*), and green (unfed + *T. cruzi*). Wild insects display more exclusive taxa; insectary samples show more overlap.

Throughout the digestive tract of *T. pallidipennis*, we identified 8 bacterial phyla and 91 genera. The phyla included Acidobacteriota, Actinobacteria, Bacteroidota, Campylobacterota, Desulfobacterota, Firmicutes, Proteobacteria, and Synergistota. Proteobacteria represented 84.3% of the reads and was present in all samples, followed by Actinobacteria with 10.57% of the reads, detected in 64 of the samples (Figure S1). The most abundant genus was *Arsenophonus* (Proteobacteria) in 40.77% of total reads, standing out as the dominant genus in insectary samples (87.11%). *Acinetobacter* (Proteobacteria) was the dominant genus in wild samples, comprising 50.23% of the total reads in this group, but only 7.69% in insectary samples. Overall, *Acinetobacter* was the second-most abundant genus across all samples (30.55% of total reads; Figure 1A).

### 3.3. Gut Microbiota Features of Insects from Wild Environments

In wild *T. pallidipennis* the gut bacterial community was primarily composed of Proteobacteria (68.94%) and Actinobacteriota (26.60%), with Firmicutes, Bacteroidota, and Synergistota present in smaller proportions. At the genus level, *Acinetobacter* was the most abundant (47.56%), followed by *Pseudomonas* (9.08%), *Rhodococcus* (7.96%), *Tsukamurella* (5.41%), and *Janthinobacterium* (4.98%) (Figure 1A and Figure S1).

In these wild insects, PCoA and PERMANOVA analyses indicated that neither feeding status nor *T. cruzi* infection had a statistically significant effect on bacterial composition (Table S2). A total of 54 bacterial genera were identified, with 48 shared between unfed and fed individuals (Figure 1C,D; Table S5). Four genera—*Pseudaminobacter*, *Altererythrobacter*, *Azotobacter*, and *Lautropia*—were exclusive to unfed insects. *Azotobacter* and *Lautropia* spanned the entire digestive tract, while *Pseudaminobacter* and *Altererythrobacter* were restricted to the stomach and intestine (Figure S2).

Feeding was not linked to specific genera, except in *T. cruzi*-infected insects where *Budvicia* appeared only in unfed individuals (Figure 1D, Table S5). Several genera, such as *Achromobacter*, *Moraxella*, *Proteus*, and *Fructilactobacillus*, were found only in fed insects, some of which had with specific distributions in different gut regions (Figure S2).

**Figure 2:**
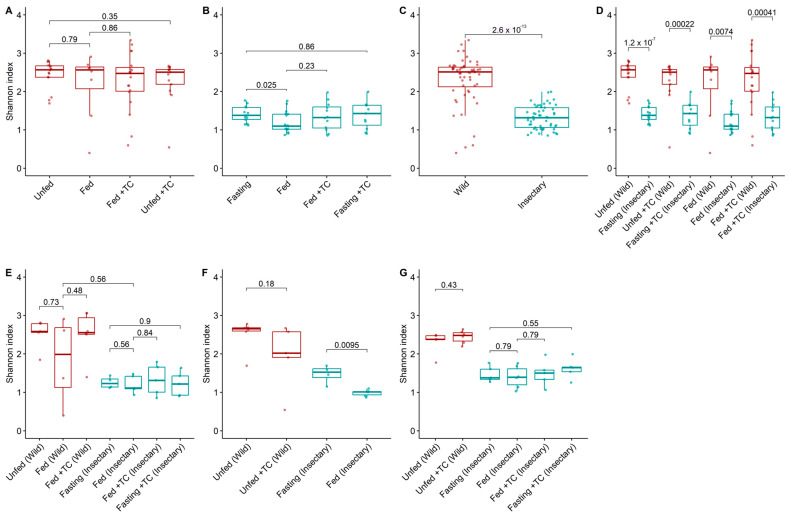
Bacterial diversity (Shannon index) in the gut microbiota of *T. pallidipennis* from different origins and physiological conditions. (**A**,**B**) Alpha diversity across experimental conditions in wild (**A**) and insectary (**B**) insects. With colours representing insect origin: red for wild and blue for insectary samples. Wild insects exhibited consistently higher diversity (Wilcoxon test), particularly under fasting conditions. (**C**) Overall comparison between wild and insectary samples. Diversity was higher in wild individuals (Wilcoxon, *p*-value < 0.001), reflecting broader environmental exposure. (**D**) Pairwise comparisons between wild and insectary groups under matched conditions. Insectary samples have consistently reduced diversity, regardless of feeding or infection status. (**E**–**G**) Diversity patterns in the stomach (**E**), intestine (**F**), and rectum (**G**) across all conditions. In all gut regions, wild insects maintain greater microbial diversity, while insectary groups display reduced diversity and higher intra-group uniformity. The largest differences were observed in the intestine (*p*-value = 0.0095) and stomach. In all panels, colour represents sample origin: red for wild, blue for insectary. For each comparison the Wilcoxon test was applied for non-parametric data (wild and cross-origin comparisons), and Student’s *t*-test for normally distributed data (insectary-only comparisons), as determined by a Shapiro–Wilk normality test.

**Figure 3:**
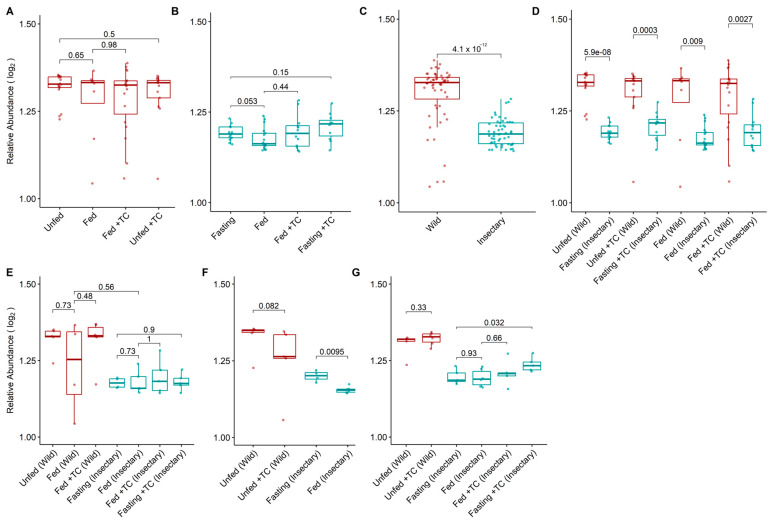
Log_2_-transformed relative abundance of gut bacterial genera in the microbiota of *T. pallidipennis* from different origins and physiological conditions. (**A**,**B**) Relative abundance across experimental conditions in wild (**A**) and insectary (**B**) insects. Colours represent sample origin: wild (red) and insectary (blue). Abundance was generally higher in wild insects (Wilcoxon test), particularly under fasting. (**C**) Overall comparison between wild and insectary samples confirms significantly higher abundance in wild individuals. (**D**) Matched comparisons across origin shows consistent reductions in abundance in insectary conditions. (**E**–**G**) Abundance patterns by gut region: stomach (**E**), intestine (**F**), and rectum (**G**). Significant differences (Student’s *t*-test) were observed in insectary insects, particularly between fasting and fed conditions in the intestine, and across multiple comparisons in the rectum. Data distribution was evaluated using the Shapiro–Wilk test to select appropriate statistical methods.

**Figure 4:**
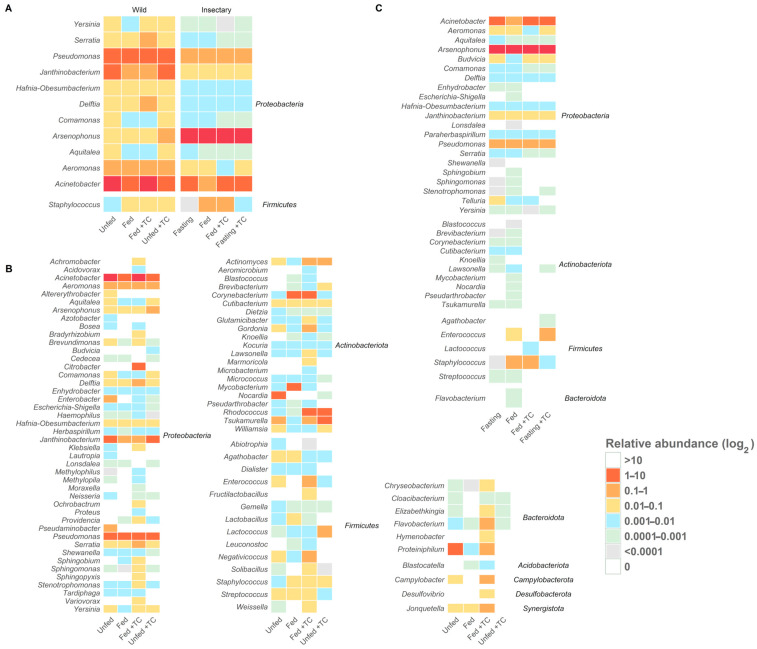
Core and origin-specific bacterial genera under different conditions in *T. pallidipennis*. Heatmaps show the log_2_-transformed relative abundance of gut bacterial genera under different origins and physiological conditions. (**A**) Core genera (twelve genera) are consistently present across all conditions, highlighting a stable microbiota component. (**B**) Insectary-specific genera show limited diversity and minor variation between conditions. (**C**) Wild-specific genera display greater diversity and marked compositional shifts with feeding and *T. cruzi* infection, indicating a more responsive and adaptable microbiota. Overall, wild insects host a more diverse and flexible community, whereas insectary insects maintain a simplified and stable profile.

**Figure 5:**
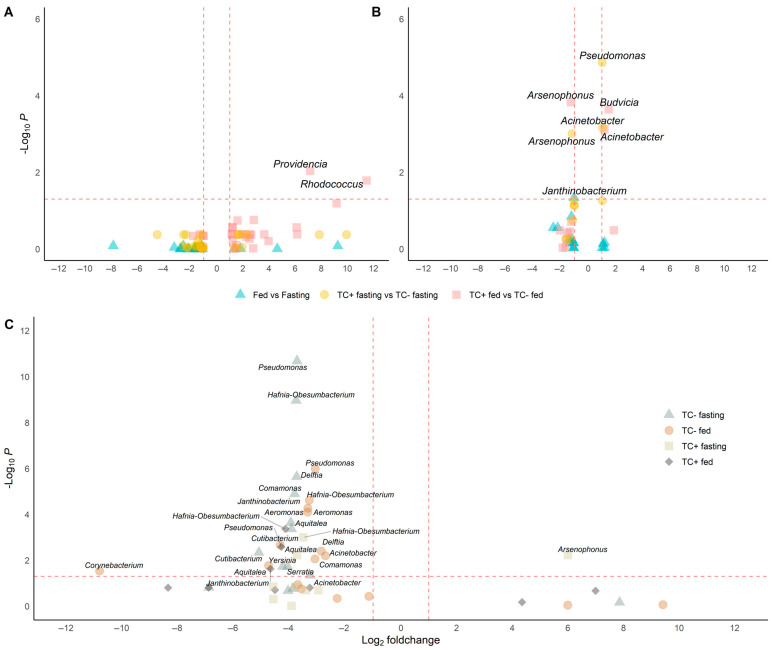
Differential abundance analysis under different conditions in wild and insectary insects. Genera with significantly different abundances are identified through pairwise comparisons using Wilcoxon or *t*-tests, depending on data distribution (Shapiro test) and including only genera detected in at least three replicates per condition. Only genera with adjusted *p*-values below 0.05 and absolute log_2_-fold changes greater than 1 are considered significant. Comparisons are visualised as volcano plots with point shapes representing specific contrasts: ▲ for Fed vs. Fasting, ● for TC + fasting vs. TC – fasting, and ■ for TC + fed vs. TC – fed. Dashed lines indicate the thresholds for significance: a horizontal line at –log_10_(*p*-value adjust ≤ 0.05) and vertical lines at log_2_-fold change = ±1. (**A**) In wild samples, *Rhodococcus* and *Providencia* are significantly more abundant in *T. cruzi*-positive fed insects. (**B**) In insectary samples, five genera show significant changes: *Acinetobacter* and *Janthinobacterium* increase in TC+ fasting, *Budvicia* in TC+ fed, and *Pseudomonas* in fed versus fasting insects. Notably, *Arsenophonus* decreases in TC+ fasting insects compared to TC−, indicating a negative association with infection under fasting in controlled conditions. (**C**) In cross-origin comparisons, genera such as *Pseudomonas*, *Aeromonas*, *Delftia*, and *Cutibacterium* are more abundant in wild insects under fasting, reflecting higher environmental exposure. Conversely, *Arsenophonus* is more abundant in insectary insects under the same TC + fasting condition compared to wild ones.

Bacterial diversity and abundance were similar between conditions (Figure 2A and Figure 3A), though the relative abundance of specific genera varied. *Acinetobacter* remained dominant (5.3–11%), while *Pseudomonas* and *Janthinobacterium* were consistently present. Other genera like *Aeromonas*, *Delftia*, and *Serratia* appeared in all conditions at lower levels (<0.3%) (Figure 4B). Notably, Providencia and *Rhodococcus* were significantly more abundant in fed *T. cruzi*-infected insects (Figure 5A).

Co-occurrence network analysis revealed condition-dependent patterns (Figure S3). In fasting uninfected insects, seven modules were detected, with a dominant cluster composed by *Pseudomonas*, *Janthinobacterium*, *Aeromonas*, and *Delftia* (Figure S3A). In fed uninfected insects, clustering became denser, involving tightly linked genera like *Pseudarthrobacter*, *Sphingomonas*, and *Leuconostoc* (Figure S3B). In fed *T. cruzi*-infected insects, modularity increased, forming distinct clusters such as those conformed by *Comamonas*–*Aquitalea*–*Yersinia* and *Pseudomonas*–*Janthinobacterium*–*Aeromonas* (Figure S3C). Fasting infected insects exhibited well-defined modules, including associations between *Ochrobactrum*, *Kocuria*, and *Tardiphaga* (Figure S3D). Overall, feeding enhanced bacterial connectivity, while *T. cruzi* infection promoted network modularity.

Functional enrichment revealed broad metabolic capabilities among bacterial clusters. Cluster with pathways like photosynthesis, fatty acid degradation, and amino acid metabolism were enriched under wild conditions. Infection and feeding further enriched clusters with capabilities for streptomycin biosynthesis and glycolysis/gluconeogenesis (Figure S5A). Z-score analysis highlighted cluster’s metabolic shifts: fasting enhanced amino acid metabolism and photosynthesis, while feeding upregulated glycolysis and fatty acid degradation. Infection boosted clusters having streptomycin biosynthesis and amino acid catabolism capabilities (Figure S5B).

Finally, differential pathway abundance (*p*-value adjust ≤0.05) showed that feeding enhanced clusters with nitrogen and carbohydrate metabolism (e.g., arginine and proline metabolism), while infection promoted clusters with glycan degradation and lysosomal pathways, indicating microbial adaptation to host and dietary changes (Figure S5C).

### 3.4. Gut Microbiota Features of Insectary Insects

Insectary insects exhibited a dominant microbiota, largely composed of Proteobacteria (94.38%), primarily *Arsenophonus* (90.45%). Other genera like *Acinetobacter* (5.57%), *Staphylococcus* (1.77%), *Pseudomonas* (0.84%), and *Enterococcus* (0.70%) were present in lower proportions (Figure 1A).

In these insectary insects, PERMANOVA analysis revealed significant differences in microbial composition across gut regions (*p*-value = 0.0334), while *T. cruzi* infection and feeding had no significant effect (Table S3). A total of 37 genera were detected, and *Paraherbaspirillum* and *Telluria* were exclusive of insectary samples. Fasting insects harboured *Shewanella* and *Knoellia*, while fed ones had unique genera like *Escherichia*-*Shigella*, *Lonsdalea*, *Sphingobium*, and *Flavobacterium* (Table S5; Figure S2). Several genera were absent in *T. cruzi*-infected individuals, including *Enhydrobacter*, *Sphingomonas*, and *Streptococcus*.

Among insectary samples, bacterial diversity was higher in fasting than in fed insects (*p*-value = 0.025; Figure 2B), with the intestine showing significant differences in both diversity and abundance (*p*-value = 0.0095; Figure 2F and Figure 3F). Fasting increased *Acinetobacter* and *Aquitalea* abundances, while feeding favoured *Sphingomonas*, *Stenotrophomonas*, *Lawsonella*, and *Staphylococcus* (Figure S2).

*Arsenophonus* remained consistently abundant across all conditions. Other genera were variable and generally had low abundance, with *Janthinobacterium* and *Paraherbaspirillum* present at low but stable levels (Figure 4C).

Differential abundance analysis showed that feeding reduced *Janthinobacterium*, while *T. cruzi* infection increased *Budvicia* and *Acinetobacter* and decreased *Arsenophonus* (Figure 5B).

Co-occurrence networks revealed condition-specific structures (Figure S4). Fasting uninfected insects had two clusters, including a negative correlation with *Arsenophonus*. Feeding led to fully positive, well-defined clusters. Infected fed insects displayed three clusters, with antagonism between *Arsenophonus* and *Acinetobacter*. Under fasting with *T. cruzi* infection, the network was minimal and dominated by a tightly linked trio: *Pseudomonas*, *Janthinobacterium*, and *Aeromonas*.

### 3.5. Differences in the Gut Microbiota Between Insectary and Wild Insects

The microbiota differed markedly between wild and insectary-reared *T. pallidipennis*. Insectary-reared insects were dominated by Proteobacteria (94%), primarily *Arsenophonus* (90%), while wild individuals had a more diverse composition, with Proteobacteria (69%), Actinobacteriota, and Firmicutes. At the genus level, wild insects showed greater variety, led by Acinetobacter (48%) and Pseudomonas (9%) (Figure 1A).

PCoA and PERMANOVA analyses showed that insect origin was the strongest factor influencing microbial composition, explaining 48.5% of the variation (*p*-value < 0.001; Figure 1B, Table S4). *T. cruzi* infection had a minor but significant effect (1.3%, *p*-value = 0.0418), while feeding (*p*-value = 0.2839) and gut region (*p*-value = 0.2489) had no significant influence, explaining only 1.1% and 1.2% of the variation, respectively (Figure 1B, Table S4).

Thirty-five genera were shared between environments, with twelve genera—Acinetobacter, Aeromonas, Aquitalea, Arsenophonus, Comamonas, Delftia, Hafnia-Obesumbacterium, Janthinobacterium, Pseudomonas, Serratia, Staphylococcus, and Yersinia—present in all gut regions, regardless of origin, feeding, or infection status (Figure 4A and Figure S2; Table S5).

Wild insects exhibited significantly higher bacterial diversity (median H′ = 2.51 vs. 1.31; *p*-value < 0.001) and abundance (1.32 vs. 1.18; *p*-value < 0.001) than insectary ones (Figure 2C and Figure 3C). This trend held across all matched conditions (Figure 2D and Figure 3D).

Differential abundance analysis showed lower levels of ten genera—including *Pseudomonas*, *Janthinobacterium*, *Delftia*, and *Acinetobacter*—in insectary-fed, uninfected insects compared to their wild counterparts (Figure 5C).

### 3.6. Inferences and Enrichment of Cluster Functional Profiles

The KEGG pathway analysis of insectary samples showed that only fasting uninfected insects displayed clusters with bacterial chemotaxis, oxidative phosphorylation, and the Citrate cycle (TCA cycle) (Figure S5A). Z-score analysis confirmed preeminent clusters with TCA cycle (Z = 1.67) and oxidative phosphorylation (Z = 2.47) under these conditions (Figure S5B). Under feeding conditions, clusters with lysine and aromatic amino acid biosynthesis, as well as DNA replication and homologous recombination were detected. Infected fasting insects showed an increase in clusters with amino sugar and fatty acid metabolism.

Comparative enrichment analysis (*p*-value adjust ≤ 0.05; Figure S5C) revealed that feeding upregulated clusters with pathways involved in bacterial cell wall and membrane biosynthesis. Feeding without infection enhanced galactose metabolism, PTS, and ABC transporters’ clusters. Infection under fasting conditions enriched clusters with sugar metabolism and structural pathways, including peptidoglycan, teichoic acid, and starch and sucrose metabolism.

Co-occurrence networks reflected disparities according to the origin of the insect samples. Wild insects formed more complex and stable networks, even under infection, whereas insectary-reared insects showed simpler, more condition-sensitive associations. Feeding increased connectivity in both groups, but only wild insects maintained modularity with infection (Figure S3; Table 1).

KEGG functional analysis revealed a shared core of enriched bacterial cluster pathways in both groups, including amino acid biosynthesis, ribosome function, protein export, and DNA repair (Figure S5A).

Wild insects, however, displayed a broader functional profile. Fasting enhanced the presence of clusters with energy metabolism (e.g., TCA cycle), while feeding and infection enriched clusters with glycolysis, streptomycin biosynthesis, and pathways linked to environmental adaptation. In contrast, insectary-reared insects showed condition-specific activation of clusters with energy-related or biosynthetic pathways, but less overall functional diversity (Figure S5B).

Differential KEGG pathway analysis (*p*-value adjust ≤ 0.05) showed that feeding in insectary-reared insects had clusters with enhanced cell wall and membrane biosynthesis, while infection under fasting promoted sugar metabolism. In wild insects, feeding induced clusters with nitrogen and carbohydrate metabolism, and infection triggered glycan degradation and lysosomal functions—reflecting environment- and condition-specific microbial adaptations (Figure S5C).

## 4. Discussion

Triatomines exhibit a lower diversity of bacterial genera in their gut microbiota compared to other insects. However, significant differences occur between triatomine species, even when they originate from similar environments [112,113], suggesting that factors beyond habitat influence these microbiota variations. In line with this, triatomine microbiomes tend to show relatively low alpha but high beta diversities [114], indicating substantial variation in microbial composition between populations. These patterns may result from a combination of environmental factors and host physiology [3,4]. The comparatively low diversity may be attributed to their obligate hematophagy, which provides a nutrient-limited diet that restricts microbial colonisation. Additionally, their compartmentalised guts and reliance on symbionts reduce the ecological niches available for broader microbial diversification.

In this study we characterised the bacterial communities of *T. pallidipennis* using 16S rRNA sequencing, enabling a broader detection of microbial taxa compared to traditional culture-based methods [115]. Our analyses allowed the identification of bacterial genera under different conditions, and the evaluation of how physiological state and *T. cruzi* infections may modulate the structure and possible function of the gut microbiota. In addition, we inferred potential ecological interactions among bacterial taxa, allowing us to propose functional hypotheses about the role of microbial communities in the insect biology. We present an integrated interpretation of (a) the microbiota’s response to the transition from a wild environment to a controlled insectary setting, (b) an interpretation of microbial adaptations to the fluctuating conditions of the digestive tract influenced by blood feeding and *T. cruzi* infection, (c) a description of a “core microbiome”, represented by bacterial taxa present in all specimens, regardless of biological or environmental conditions, and (d) a set of bacterial taxa potentially participating in *T. pallidipennis* physiology. Our findings help to understand the complex interactions between host, microbiota, and pathogen, and set the stage for future functional and ecological analyses.

Although PERMANOVA did not detect significant effects of feeding status or *T. cruzi* infection on overall community structure, the differential abundance and functional analyses showed that each factor, when evaluated independently, influence specific bacterial genera and metabolic pathways. This highlights that feeding and infection can shape the microbiota at a more detailed taxonomic and functional level, even when global diversity appears unchanged.

We identified ninety-one bacterial genera in eight phyla: with Proteobacteria with 51% of the bacterial genera identified. The prominent presence of Proteobacteria appears to be conserved across species of the *Triatoma* and *Rhodnius* genera [116,117,118]. Despite variations, some bacterial genera were present in all specimens analysed, regardless of their origin, nutritional status, or infection. This stable set constitutes the “core microbiota”, composed of twelve genera: *Acinetobacter*, *Aeromonas*, *Aquitalea*, *Arsenophonus*, *Comamonas*, *Delftia*, *Hafnia-Obesumbacterium*, *Janthinobacterium*, *Pseudomonas*, *Serratia*, *Yersinia* (Proteobacteria), and *Staphylococcus* (Firmicutes). Their presence in both wild and insectary insects indicates that their success depends more on compatibility with the gut environment than on external factors. Their persistence may be explained by their rapid response to physiological stress, including oxidative pressure and nutrient shifts following blood feeding [48,49].

Previous work, using PCR-DGGE, reported *Bacillus* and *Staphylococcus* as dominant genera in *Meccus* (*Triatoma*) *pallidipennis* and found minimal differences between wild and laboratory insects (the latter fed on mouse blood) [119]. In contrast, our study applied high throughput 16S rRNA sequencing to insects from southern Morelos maintained on rabbit blood, revealing *Arsenophonus* and *Acinetobacter* as the predominant genera and marked differences between wild and insectary groups. The sequencing approach allowed us to detect a broader diversity of both culturable and unculturable bacteria, providing a more comprehensive profile of the gut microbiota and uncovering differences that may be missed by culture-dependent techniques. These results show that both methodological approaches and differences in diet or locality can significantly affect the observed composition of the gut microbiota.

The composition and structure of the gut microbiota were most strongly influenced by the insect’s origin, followed by nutritional status, and then by the presence of *T. cruzi*.

The shift from a natural to a controlled environment highlights the following two key microbial dynamics: some genera, likely acquired through continuous exposure to environmental sources, tend to disappear under laboratory conditions; while others, once established in the wild, persist in the insectary, suggesting that these are better suited to colonising the host or adapting to more simplified and stable gut environments. Although PERMANOVA did not detect significant effects of feeding status or *T. cruzi* infection on overall community structure, our differential abundance and functional analyses showed that both factors influence specific bacterial genera and metabolic pathways. This highlights that feeding and infection can shape the microbiota at a more detailed taxonomic and functional level, even when global diversity appears unchanged.

Genera like *Flavobacterium*, *Cutibacterium*, and *Rhodococcus*, which are common in wild insects and associated with xenobiotic degradation, nitrogen fixation, or antimicrobial activity [54,60,90], were absent or significantly reduced in the insectary. These taxa likely require constant replenishment to persist, suggesting they represent transient colonisers maintained through repeated reinfection in natural settings. Conversely, vertically transmitted or stress-tolerant genera such as *Arsenophonus*, *Acinetobacter*, and *Pseudomonas* were consistently maintained, reflecting their ability to colonise and adapt within the more restricted conditions of the insectary [90,103].

In wild insects microbial diversity was notably higher, likely due to prolonged and repeated exposure to diverse sources during development. Although triatomines are generalist blood-feeders, extended contact with natural substrates facilitates the acquisition of a broader range of bacterial taxa. Blood feeding introduces a complex nutrient profile that reshapes microbial composition, creating metabolic niches that favour bacteria capable of processing these substrates. For example, an enrichment of *Budvicia*, involved with fermentation processes; *Lactobacillus* and *Enterobacter*, involved in protein and carbohydrate digestion; and *Flavobacterium* and *Sphingobium*, which participate in the detoxification of blood-derived compounds [54,90,95]. This shift reflects microbial adaptation to the transient and intense metabolic demands imposed by hematophagy. Under fasting conditions, other metabolic demands need to be met. The increase in *Acinetobacter*, *Aeromonas*, and *Pseudomonas* may be related to detoxification processes and resilience to environmental changes [49,54,90,120].

In insects reared in insectaries microbial diversity was initially low, primarily due to controlled environmental and dietary conditions, as well as reduced exposure to diverse microbial sources. However, feeding also had a marked impact on the microbiota. *Acinetobacter* and *Janthinobacterium* declined as their adaptation to low-nutrient, high-stress conditions offers less advantage in the nutrient-rich gut environment created by blood feeding. This favoured fast-growing, specialised taxa such as *Budvicia*, *Flavobacterium*, and *Sphingobium*, capable of metabolising blood components and detoxifying xenobiotics [90,95,105]. Under fasting condition, the microbiota was dominated by *Arsenophonus*, with the co-occurrence of bacteria involved in detoxification (*Pseudomonas*, *Aeromonas*, *Delftia*) and stress adaptation (*Janthinobacterium*, *Comamonas*) [51,102].

Although *T. cruzi* infection explained a small proportion of microbial variation, its effects were statistically significant. *T. cruzi* had dissimilar effects in wild and insectary insects that could be explained by similar alterations in the gut milieu. In wild insects, the presence of *T. cruzi* and blood feeding consistently favoured *Serratia*, *Providencia*, and *Rhodococcus,* as previously observed [60,109,121,122]. This shift likely reflects a response to parasite-induced changes in the gut environment, including tissue damage, immune activation, and the competition for nutrients, favouring stress-tolerant or immunologically interactive taxa. In the insectary, blood feeding of *T. cruzi*-infected bugs produced an increase in *Pseudomonas*, *Acinetobacter*, and *Serratia,* which are bacteria known to interfere with parasite survival in other vectors [108,109]. A sharp decline in *Arsenophonus* likely resulted from infection-triggered immune responses and epithelial stress, which destabilise the gut environment leading to a disadvantage for this symbiont [123,124] alongside the expansion of competitive or antagonistic taxa. Fasting in the presence of *T. cruzi* led to the emergence of *Yersinia*, *Aquitalea*, and *Telluria*, suggesting an immune- or parasite-mediated modulation [52,53]. In this context, *T. cruzi* infection not only reduced microbial diversity—even without feeding—but also reconfigured the community by imposing selective pressures driven by tissue damage, immune shifts, and metabolic competition. Blood feeding of wild and insectary insects favoured genera such as *Budvicia*, *Flavobacterium*, and *Sphingobium* as well as *Serratia* in the presence of *T. cruzi*.

Members of the “core microbiota” are widely recognised for their colonisation abilities, resistance to adverse conditions, and possible interactions with the host immune system [18,117]. Their persistence across conditions implies a functional importance in digestion, pathogen defence, and gut homeostasis. These genera have been identified in other triatomine species, indicating a potentially conserved symbiotic relationship across different hosts and environmental conditions [113,125]. Several members of this core, such *Acinetobacter*, *Aeromonas* and *Pseudomonas*, contribute to iron acquisition and detoxification, supporting survival under fasting and blood digestion stress [70,90,95]. *Arsenophonus*, dominant in insectary insects, likely compensates for microbial scarcity through B-vitamin synthesis [103], though its decline with *T. cruzi* infection suggests parasite-driven modulation [108]. *Comamonas*, *Delftia*, and *Aquitalea* provide D-amino acids [51]; *Janthinobacterium* can modulate microbial communities via violacein [52]; and *Serratia* may inhibit parasite colonisation by producing antimicrobial lipodepsipeptides [109]. Although less abundant, *Yersinia* and *Staphylococcus* may persist due to their membrane properties that provide resistance to host defences [107]. While not obligate symbionts, these genera appear indispensable under fluctuating physiological conditions, creating a resilient microbiota that contributes to host survival and may influence vector competence.

Supporting this functional relevance, KEGG pathway analysis revealed that several core taxa contribute to pathways associated with protein synthesis and genome maintenance. This suggests that microbial diversity contributes to a metabolically stable gut environment under nutritional stress [54,60]. In particular, *Acinetobacter*, *Pseudomonas*, *Aeromonas*, *Arsenophonus*, *Comamonas*, *Delftia*, *Serratia*, and *Staphylococcus* appear to share core metabolic roles [49,51,95,109], including aminoacyl-tRNA biosynthesis, protein export, lysine biosynthesis, peptidoglycan biosynthesis, and protein synthesis, as well as cell wall maintenance.

The co-occurrence networks analysis revealed distinct patterns of bacterial interactions depending on the physiological state and infection status. Under fasting conditions networks displayed a more modular and less connected structure, suggesting more specialised, stable communities. In contrast, networks in blood-fed and infected insects were more complex and densely connected, with multiple positive associations between taxa indicating a more dynamic and interdependent community.

These co-occurrence patterns may reflect potential ecological interactions such as symbiosis, competition, or commensalism. Our results revealed that both physiological state and *T. cruzi* infection profoundly modulate the structure and potential function of the gut microbiota. Positive and negative correlations observed between bacterial genera suggest mutualism, niche overlap, competitive exclusion, or antagonism. In particular, fasting insectary insects free of *T. cruzi* harboured bacteria associated with detoxification (*Pseudomonas*, *Acinetobacter*, *Aeromonas*), nutritional support under stress (*Arsenophonus*), and microbial or developmental regulation (*Delftia*, *Comamonas*, *Janthinobacterium*) [49,51,102]. In contrast, fed individuals showed a shift toward functions related to iron acquisition, fermentation, and xenobiotic degradation, processes often linked to bacteria from the genera *Pseudomonas*, *Budvicia*, *Flavobacterium*, and *Sphingobium* [49,95,105].

Wild specimens, by contrast, exhibited more complex and positively correlated networks, implying greater microbial cooperation and specialisation—particularly in nitrogen metabolism, antimicrobial production, and modulation of host defences. In the unfed situation the microbiota favours detoxification and stress adaptation, with *Acinetobacter*, *Aeromonas*, and *Pseudomonas* playing important roles in bioremediation and toxin degradation [49,54,90,120]. Blood feeding, however, promotes metabolic diversity, incorporating bacteria involved in fermentation (*Budvicia*), iron uptake (*Acinetobacter*, *Aeromonas*), and xenobiotic degradation (*Flavobacterium*, *Sphingobium*) [95,106]. This shift may reflect an adaptive response favouring synergistic bacterial interactions that enhance nutrient processing and resilience to stressors [126,127].

By integrating taxonomic profiles, functional predictions, and network analyses, we can propose the ecological functionalities of several taxa. For example, *Serratia* and *Providencia* may participate in direct competition with *T. cruzi*, while *Acinetobacter* and *Comamonas* may be involved in the degradation of toxic compounds derived from blood digestion. These proposed functions can be validated experimentally using transcriptomics or culture-based assays. Manipulating the microbiota through probiotics or antibiotics may allow for evaluation of their participation in infection and vector physiology, opening new opportunities for biological control strategies. Similar microbiota-based strategies have shown promise in other disease vectors, such as the use of *Wolbachia* in mosquitoes to inhibit arboviral replication [128] or *Enterobacter* strains that block *Plasmodium* in *Anopheles* [108]. Resident taxa like *Serratia*, *Pseudomonas*, and *Comamonas* represent promising targets for microbiome-based strategies aimed at reducing triatomine vector competence.

This study provides a comprehensive characterisation of the gut microbiota of *T. pallidipennis* using high-throughput 16S rRNA sequencing; however, several methodological limitations should be acknowledged. The identification of bacteria was restricted to the genus level as 16S rRNA sequencing does not reliably resolve species- or strain-level diversity. In addition, amplification bias may influence the apparent abundance of particular taxa, and this approach does not provide absolute quantification, thereby limiting ecological interpretation of microbial loads. Furthermore, while bacteria with atypical or highly divergent rRNA genes may go undetected—a recognised limitation of this technique—our data demonstrate that key intracellular symbionts such as *Arsenophonus* were successfully identified and profiled. Thus, although some rare or atypical taxa may remain undetected, our approach captured both free-living and important intracellular bacteria relevant to the *T. pallidipennis* microbiota.

Despite these limitations, the use of 16S rRNA sequencing enabled the profiling of both culturable and unculturable bacteria and revealed patterns that would not be accessible with traditional culture-based methods. Future research should address these limitations by including broader temporal and geographical sampling, direct functional assays, and complementary methods such as metagenomics or culture-based techniques.

## 5. Conclusions

Our results demonstrate that the gut microbiota of *T. pallidipennis* is structured by a combination of environmental (e.g., insect origin) and physiological factors (e.g., feeding and infection), and that some key bacterial taxa persist and potentially fulfil important functions in the host. We documented the ecological plasticity of the *T. pallidipennis* microbiota and suggested that its composition may influence host physiology and vector competence. These insights lay the groundwork for future studies exploring functional interactions between host, microbiota, and pathogens, and open the door to microbiota-based strategies for vector control.

## Figures and Tables

**Table 1:** Functional roles of bacterial clusters in *T. pallidipennis* across feeding and infection conditions.

Condition	Cluster	Genera	Associated Functions	References
**Wild Unfed**	Cluster 1 (C1)	*Acinetobacter*, *Aeromonas*, *Arsenophonus*, *Pseudomonas*	*Pseudomonas* and *Aeromonas* regulate the gut microbiota and degrade toxins via hydrogen cyanide, cyclic lipopeptides, and type VI secretion systems in *S*. *littoralis* and *Culex pipiens*. *Comamonas* and *Delftia* synthesise D-alanine and D-glutamate through racemases in *H*. *vitripennis*. *Janthinobacterium* inhibits *F*. *graminearum* by producing violacein regulated by the vioABCDE gene cluster.	[48,49,50,51,52,53]
	Cluster 2 (C2)	*Cutibacterium*, *Enhydrobacter*, *Lawsonella*	*Cutibacterium, Enhydrobacter*, and *Lawsonella* fix nitrogen and ferment carbohydrates via nitrogenases and dehydrogenases, reported in the gut microbiota of arthropods such as hunting spiders and marine copepods.	[54,55]
	Cluster 3 (C3)	*Altererythrobacter*, *Enterobacter*, *Glutamicibacter*, *Lactobacillus*, *Pseudaminobacter*	*Enterobacter* and *Lactobacillus* enhance carbohydrate metabolism via glycolytic and fermentation pathways and tolerate pH and temperature shifts through membrane adaptations and stress response proteins, reported in beetles and lepidopterans. *Glutamicibacter* and *Enterobacter* support environmental adaptation by activating oxidative stress pathways involving catalases and peroxidases.	[56,57,58,59]
	Cluster 4 (C4)	*Corynebacterium*, *Rhodococcus*, *Williamsia*	*Corynebacterium, Rhodococcus*, and *Williamsia* promote the development of *R. prolixus* by producing B vitamins, including biotin and riboflavin, through key biosynthetic enzymes such as *BioA/B/C/D/F* and *RibA/B/D/E*, aiding host growth under nutrient stress.	[60,61]
	Cluster 5 (C5)	*Actinomyces*, *Campylobacter*, *Jonquetella*, *Negativicoccus*, *Proteiniphilum*	*Actinomyces*, *Campylobacter*, *Jonquetella*, *Negativicoccus*, and *Proteiniphilum* degrade complex organic compounds via hydrolases and anaerobic metabolic enzymes, contributing to stress tolerance in the insect gut. Specifically, *Proteiniphilum* and *Actinomyces* produce antimicrobial compounds, observed in *Helicoverpa armigera* and *Methanothrix*-dominated systems under ammonia stress.	[62,63]
	Cluster 6 (C6)	*Azotobacter*, *Gemella*, *Lautropia*, *Neisseria*	*Azotobacter* fixes atmospheric nitrogen via nitrogenase, reported in the rhizosphere and insect gut environments. *Neisseria and Gemella* reduce nitrate through nitrate reductases, documented in the intestinal microbiota of spiders (*Thomisidae* and *Oxyopidae*).	[64,65]
	Cluster 7 (C7)	*Kocuria*, *Micrococcus*, *Pseudarthrobacter*, *Tardiphaga*	*Kocuria* and *Micrococcus*, from *Aedes aegypti*, produce volatile organic compounds (VOCs) via fatty acid and amino acid catabolism, acting as semiochemicals that influence host behaviour. *Pseudarthrobacter* and *Tardiphaga*, from *H. armigera*, degrade flonicamid through xenobiotic pathways (benzoate, toluene, aminobenzoate) using monooxygenases, dioxygenases, aldehyde dehydrogenases, and amidases.	[66,67]
**Wild—Fed**	Cluster 1 (C1)	*Blastocatella*, *Leuconostoc*, *Pseudarthrobacter*, *Sphingomonas*	*Pseudarthrobacter* and *Sphingomonas*, isolated from *H. armigera*, degrade insecticides like flonicamid through xenobiotic catabolic pathways (e.g., benzoate, aminobenzoate, toluene degradation), involving enzymes such as flavin-dependent monooxygenases, aldehyde dehydrogenases, and organophosphate hydrolases.	[67,68]
	Cluster 2 (C2)	*Acinetobacter*, *Aquitalea*, *Lactococcus*	In *Novius pumilus*, *Aquitalea* and *Lactococcus* contribute to prey digestion by degrading hydrocarbons, fatty acids, and chitin through catabolic pathways. *Acinetobacter*, known from leech gut studies, enhances survival under stress via iron acquisition systems such as acinetobactin-mediated siderophore uptake.	[69,70]
	Cluster 3 (C3)	*Chryseobacterium*, *Gemella*, *Haemophilus*, *Knoellia*	*Chryseobacterium*, isolated from *Melolontha melolontha* larvae, inhibits mutualistic bacteria of entomopathogenic nematodes and acquires iron via the citrate-based siderophore chryseochelin *A. Haemophilus* enhances respiration by modifying its electron transport chain in response to hemin, promoting growth under iron-limiting conditions.	[71,72,73]
	Cluster 4 (C4)	*Brevundimonas*, *Herbaspirillum*, *Rhodococcus*	*Brevundimonas* and *Rhodococcus*, isolated from aquatic environments, degrade insecticides such as nitenpyram via hydroxylation and organophosphate hydrolase pathways.	[74,75]
	Cluster 5 (C5)	*Comamonas*, *Cutibacterium*, *Delftia*, *Enhydrobacter*, *Lawsonella*, *Shewanella*, *Stenotrophomonas*, *Tardiphaga*, *Wilimasia*, *Yersinia*	*Comamonas* in *Aedes atropalpus* promotes host development and egg production by supporting nutrient metabolism. *Stenotrophomonas*, isolated from *B. mori*, increases biosynthesis of essential amino acids (e.g., Arg, Thr, Leu, Val, Glu) via enzymes such as acetolactate synthase and prephenate dehydratase. *Yersinia*, studied in *Galleria mellonella*, resists host antimicrobial peptides through outer membrane modifications regulated by the PhoPQ system.	[13,76,77]
	Cluster 6 (C6)	*Actinomyces*, *Jonquetella*, *Negativicoccus*, *Proteiniphillum*, *Sphingobium*	*Proteiniphilum* and *Actinomyces*, reported in insect microbiota including *S. frugiperda*, degrade complex organic matter under stress via anaerobic enzymes and produce antimicrobial compounds (polyketides, cyclic lipopeptides, and RiPPs). *Sphingobium*, also identified in *S. frugiperda*, expresses an iron-regulated organophosphate hydrolase that enables pesticide degradation under iron-limited conditions.	[62,63]
	Cluster 7 (C7)	*Aeromonas*, *Gordonia*, *Hafnia-* *Obesumbacterium*, *Janthinobacterium*, *Pseudomonas*, *Serratia*	*Pseudomonas* (in *S. littoralis*) modulates gut microbiota via type VI secretion and glycan modifications conferring resistance to antimicrobial peptides. *Aeromonas* (in *H. verbana*) acquires iron and heme under stress using ahu/hmu transporters. *Serratia* (in mosquitoes) produces antimicrobial lipodepsipeptides that impair gut colonisation and motility.	[49,70,78,79,80]
	Cluster 8 (C8)	*Cedecea*, *Dietzia*, *Kocuria*	*Cedecea* colonises the gut of *A. aegypti* by forming biofilms that support stable gut persistence. *Dietzia*, isolated from the alkaline gut of *Trypoxylus dichotomus* larvae, exhibits alkaliphilic metabolic traits, including xylanolytic activity, which supports survival under high-pH conditions.	[81,82]
**Wild—Blood-fed + *T. cruzi***	Cluster 1 (C1)	*Citrobacter*, *Elizabethkingia*, *Enterococcus*, *Klebsiella oxytoca*	*Citrobacter* contributes to nitrogen recycling and cellulose degradation via cellulases and deaminases in desert weevil larvae. *Elizabethkingia* protects *A. gambiae* from oxidative stress by producing antioxidant enzymes such as superoxide dismutase and catalase. *Enterococcus* enhances host antimicrobial defences in *G. mellonella* by inducing innate immune responses and defensin production. *Klebsiella oxytoca* promotes nitrogen metabolism and outcompetes microbes in *Ceratitis capitata* by producing colicin-like bacteriocins and nitrate reductases.	[83,84,85,86,87]
	Cluster 3 (C3)	*Bradyrhizobium*, *Herbaspirillum*, *Flavobacterium*, *Variovorax*, *Sphingopyxis*, *Microbacterium*	*Bradyrhizobium* and *Herbaspirillum* fix atmospheric nitrogen via nitrogenase activity, reported in association with tropical forage legumes. *Flavobacterium* and *Variovorax*, identified in *S. frugiperda* and agricultural soils, respectively, contribute to pesticide detoxification through xenobiotic degradation pathways. *Sphingopyxis*, isolated from various environments, performs anaerobic respiration via nitrate reduction. *Microbacterium*, associated with *Atta cephalotes*, protects the host against fungal pathogens through the secretion of antimicrobial metabolites.	[88,89,90,91,92,93,94]
	Cluster 4 (C4)	*Acinetobacter*, *Aeromonas*, *Pseudomonas*	*Acinetobacter* and *Aeromonas*, isolated from *H. verbana* and *C. pipiens*, acquire iron via siderophores and iron/heme transporters (e.g., acinetobactin, ahu/hmu). *Pseudomonas*, reported in *S. littoralis*, modulates gut microbiota through type VI secretion systems and resistance to antimicrobial peptides via glycan surface modifications.	[49,70,79,95,96]
	Cluster 6 (C6)	*Campylobacter*, *Negativicoccus*, *Proteiniphilum*	*Campylobacter*, *Negativicoccus*, *Proteiniphilum* degrade organic matter in *G. mellonella* via acetogenic fermentation pathways, producing acetate and other short-chain fatty acids through enzymes like glutamate dehydrogenase and acetyl-CoA synthetase, enabling adaptation to anaerobic and nutrient-rich gut environments.	[63]
**Wild—Unfed + *T. cruzi***	C1-C2 (C1-C2)	*Janthinobacterium*, *Aeromonas*, *Yersinia*, *Providencia*	*Janthinobacterium*, identified in *F. graminearum* interactions, produces violacein via the vioABCDE cluster, contributing to antimicrobial activity. *Aeromonas*, found in *C. pipiens*, regulates gut microbiota and detoxifies through iron uptake systems. *Yersinia*, studied in fleas, resists host antimicrobial peptides via lipid A modifications and PhoPQ-regulated responses. *Providencia*, detected in leeches, synthesises B vitamins like biotin through symbiotic genome reduction.	[50,52,97,98,99,100]
**Insectary—Fasting**	Cluster 1 (C1)	*Acinetobacter*, *Aeromonas*, *Arsenophonus*, *Pseudomonas*	*Acinetobacter* and *Aeromonas* promote survival under nutrient stress via iron acquisition using acinetobactin and ahu/hmu transporters in leeches. *Pseudomonas* modulates gut microbiota in *Spodoptera littoralis* by producing cyclic lipopeptides and hydrogen cyanide. *Arsenophonus* aids nutrition in aphids and whiteflies by synthesising biotin (bioA–F) and riboflavin (ribA–E) through horizontally transferred genes.	[42,48,49,97,101,102,103,104]
	Cluster 2 (C2)	*Aquitalea*, *Comamonas*, *Delftia*, *and Janthinobacterium*	*Comamonas* and *Delftia* promote insect development by producing D-alanine and D-glutamate in *Homalodisca vitripennis*. *Janthinobacterium* inhibits *Fusarium graminearum* by synthesising violacein via the quorum-sensing gene cluster vioABCDE.	[51,52,53]
**Insectary—Blood-fed**	Cluster 1 (C1)	*Acinetobacter*, *Aeromonas*, *Budvicia*, *Delftia*, *Hafnia-Obesumbacterium*, *Janthinobacterium*, *Pseudomonas*	*Acinetobacter* and *Aeromonas* acquire iron via acinetobactin and ahu/hmu transporters to cope with nutrient stress in *Hirudo verbana*. *Pseudomonas* modulates the gut microbiota in *S. littoralis* through T6SS effectors and surface glycan modifications for AMP resistance. *Budvicia* ferments glucose into acetic acid to aid digestion in *Rhynchophorus ferrugineus* larvae.	[49,70,79,95,96,105]
	Cluster 2 (C2)	*Flavobacterium*, *Lonsdalea*, *Pseudarthrobacter*, *Sphingobium*, *Stenotrophomonas*	*Flavobacterium* degrades insecticides in *Spodoptera frugiperda* via xenobiotic degradation pathways. *Stenotrophomonas*, from *Bombyx mori*, enhances Arg, Thr, Leu, Val, and Glu biosynthesis through enzymes like acetolactate synthase and prephenate dehydratase. *Sphingobium*, also in *S. frugiperda*, expresses iron-regulated organophosphate hydrolase under iron scarcity, breaking down pesticides.	[76,90,106]
**Insectary—Blood-fed + *T. cruzi***	Cluster 1 (C1)	*Aquitalea*, *Comamonas*, *Telluria*, *Yersinia*	*Aquitalea*, *Comamonas* (*H. vitripennis*), and *Telluria* aid insect metabolism by producing D-amino acids and ammonia via racemases and deaminases. *Yersinia pestis*, in fleas, resists antimicrobial peptides through outer membrane changes regulated by the PhoPQ system.	[51,77,107]
	Cluster 2 (C2)	*Acinetobacter*, *Arsenophonus*, *Pseudomonas*	*Acinetobacter* and *Pseudomonas* (*Anopheles gambiae*) use siderophores and T6SS to outcompete microbes and evade host immunity. *Arsenophonus* (aphids, whiteflies) synthesises B vitamins (biotin, riboflavin) via horizontally acquired genes (e.g., bioA, bioB, ribD, ribE), aiding host nutrition under stress.	[102,103,104,108]
	Cluster 3 (C2)	*Aeromonas*, *Budvicia*, *Delftia*, *Hafnia-Obesumbacterium*, *Janthinobacterium*, *Serratia*	*Serratia* (*R. prolixus*, *Anopheles stephensi*) produces lipodepsipeptides that impair gut colonisation and motility, affecting *T. cruzi* and *Plasmodium transmission*. *Pseudomonas* and *Acinetobacter* (*Anopheles gambiae*) use T6SS and glycan-modified membranes to resist antimicrobial peptides and influence *Plasmodium infection*. *Delftia* (*Anopheles*) synthesises D-amino acids (D-Ala, D-Glu) via racemases, supporting host development and potentially modulating parasite dynamics.	[52,53,109,110,111]
**Insectary—Fasting + *T. cruzi***	Cluster 1 (C3)	*Aeromonas*	*Aeromonas* (*Culex pipiens quinquefasciatus*) regulates gut microbiota and detoxifies insecticides through iron uptake systems and xenobiotic degradation pathways.	[50,97]
	Cluster 2 (C3)	*Janthinobacterium* and *Pseudomonas*	*Janthinobacterium* (*F. graminearum*) modulates microbiota via violacein synthesis regulated by the vioABCDE quorum-sensing cluster. *Pseudomonas* (*S. littoralis*) shapes gut ecology through cyclic lipopeptides, hydrogen cyanide, and T6SS, promoting microbial regulation during infection.	[48,49,52,53]

## Data Availability

Data_Supplementary.xlsx and accession temporal number: SUB15422587.

## Supplementary Materials

The following supporting information can be downloaded at: https://www.mdpi.com/article/10.3390/biology14080984/s1, Figure S1: Distribution of ASVs by abundance and prevalence in the gut microbiota of *Triatoma pallidipennis* under different physiological and infection conditions; Figure S2: Log_2_-transformed the relative abundance of bacterial genera across gut regions in *T. pallidipennis* under insectary and wild conditions; Figure S3: Co-occurrence network of the gut microbiota of *T. pallidipennis* under wild conditions; Figure S4: Co-occurrence network analysis of the gut microbiota of *T. pallidipennis* under insectary conditions; Figure S5. Functional enrichment of KEGG pathways across feeding conditions, *T. cruzi* infection, and environmental origin in *T. pallidipennis*. Table S1. Summary of sequenced samples and experimental groups; Table S2. PERMANOVA results of microbial community composition based on environmental and biological factors in wild samples. Table S3. PERMANOVA results on microbial community composition based on environmental and biological factors in insectary samples; Table S4. PERMANOVA results for microbial community composition based on environmental and biological factors. Table S5. Bacterial genera present in insectary and wild samples across analysed conditions. Supplementary data: differential abundance; network co-occurrence analyses; enrichment KEGG per condition; Z-score analysis KEGG; abundance diff_enrich_KEGG.

## Abbreviations

The following abbreviations are used in this manuscript:

ASVs	Amplicon Sequence Variants
PCoA	Principal Coordinates Analysis
FDR	False Discovery Rate
KEGG	Kyoto Encyclopaedia of Genes and Genomes
KOs	KEGG Orthologs
ORA	Over-Representation Analysis
