# Supplementary material for: Influence of the Origin, Feeding Status, and Trypanosoma cruzi Infection in the Microbial Composition of the Digestive Tract of Triatoma pallidipennis
Source: Biology (Basel). 2025 Aug 2;14(8):984. doi: 10.3390/biology14080984 (PMC12383893; doi:10.3390/biology14080984)
Supplement: Supplementary file 1 [file biology-14-00984-s001.zip › Supplementary_Files_16S Triatominae.pdf]

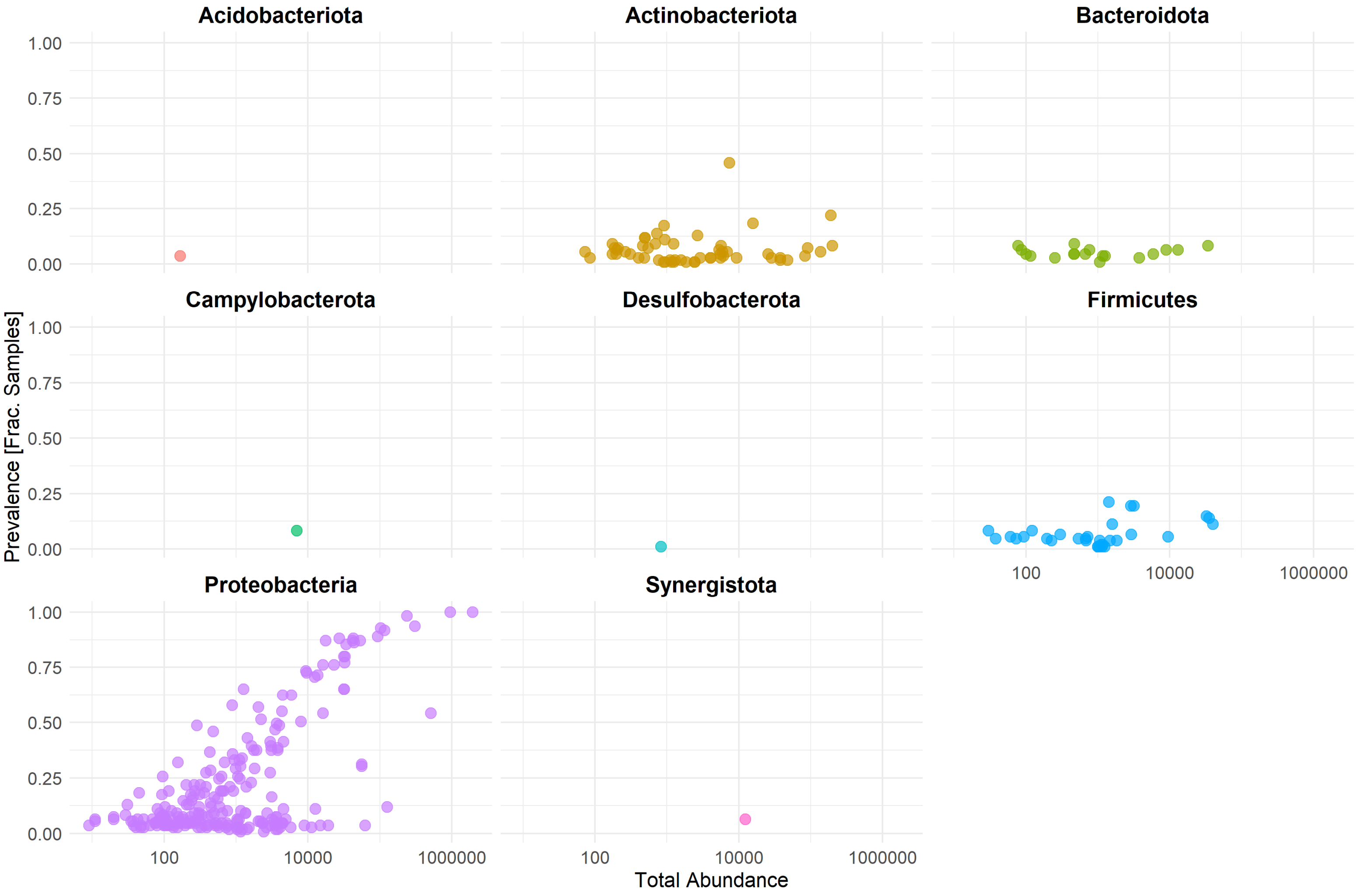

Figure S1. Distribution of ASVs by abundance and prevalence in the gut microbiota of *Triatoma pallidipennis* under different physiological and infection conditions. Total abundance represents cumulative raw read counts per ASV (x-axis, log_{10} scale), while prevalence refers to the fraction of samples in which each ASV appears (y-axis). Each point represents a unique ASV coloured by phylum. Most ASVs are low in abundance and prevalence, but some—particularly *Proteobacteria* and *Bacteroidota*—are both highly abundant and widely shared across samples, indicating a core gut microbiota. Less prevalent phyla such as *Actinobacteriota* and *Firmicutes* display sparser ASV distributions, indicating a more variable or transient role. The figure highlights the coexistence of dominant taxa and a diverse low-abundance background community.

Table S1. Summary of sequenced samples and experimental groups.

Condition	Anatomical Region	Number of Samples	Reads/Percentage
**Wild-Unfed**	Stomach	5	353206/5.33%
	Intestine	6	371089/5.60%
	Rectum	5	264553/3.99%
		**16**	**988,848/14.92%**
**Wild-Fed**	Stomach	4	279535/4.22%
	Intestine	2	156818/2.37%
	Rectum	2	167472/2.53%
		**8**	**603,825/9.11%**
**Wild-Blood-fed + *T. cruzi***	Stomach	6	373861/5.64%
	Intestine	6	447426/6.75%
	Rectum	7	469266/7.08%
		**19**	**1290,553/19.48%**
**Wild-Unfed + *T. cruzi***	Stomach	2	151224/2.28%
	Intestine	5	370511/5.59%
	Rectum	6	318709/4.81%
		**13**	**840,444/12.68%**
**Insectary-Fasting**	Stomach	4	316873/4.78%
	Intestine	4	272840/4.12%
	Rectum	5	343211/5.18%
		**13**	**932,924/14.08%**
**Insectary-Blood-fed**	Stomach	5	459640/6.94%
	Intestine	6	470594/7.10%
	Rectum	6	459666/6.94%
		**17**	**1,389,900/20.97%**
**Insectary-Blood-fed + *T. cruzi***	Stomach	5	144729/2.18%
	Intestine	2	38310/0.58%
	Rectum	5	140831/2.13%
		**12**	**323,870/4.89%**
**Insectary-Fasting + *T. cruzi***	Stomach	5	135089/2.04%
	Intestine	1	26767/0.40%
	Rectum	5	94363/1.42%
		**11**	**256,219/3.87%**
	**Total**	**109**	**6,626,583/100%**

Table S2. PERMANOVA results of microbial community composition based on environmental and biological factors in wild samples.

Factor	Sum of squares	R^2	*F*	*p-value*
T. cruzi	0.0669	0.00896	0.4836	0.7302
Feeding	0.1477	0.01980	1.0681	0.3244
Organ	0.1936	0.02596	0.7002	0.6363
**Residual**	7.0515	0.94528		
**Total**	7.4596	1.00000		

Significance codes: **0.001 '***', 0.01 '**', 0.05 '*'**.

Note: R^2 indicates the proportion of total variance in microbial composition explained by each factor. The *F value* (pseudo-F) reflects the degree of compositional differences between groups defined by that factor; higher values suggest stronger group separation.

Table S3. PERMANOVA results on microbial community composition based on environmental and biological factors in insectary samples.

Factor	Sum of squares	R^2	*F*	*p-value*
T. cruzi	0.03064	0.02844	1.5690	0.2071
Feeding	0.02767	0.02569	1.4171	0.2697
Organ	0.08149	0.07566	2.0866	**0.0334** *
**Residual**	0.93724	0.87021		
**Total**	1.07704	1.00000		

Significance codes: **0.001 '***', 0.01 '**', 0.05 '*'**.

Note: R^2 indicates the proportion of total variance in microbial composition explained by each factor. The *F value* (pseudo-F) reflects the degree of compositional differences between groups defined by that factor; higher values suggest stronger group separation.

Table S4. PERMANOVA results for microbial community composition based on environmental and biological factors.

Factor	Sum of squares	R^2	*F*	*p-value*
T. cruzi	0.2229	0.01317	2.8081	**0.0418** *
Type	8.2165	0.48547	103.5319	**0.0001** ***
Feeding	0.1902	0.01124	1.1982	0.2839
Organ	0.2003	0.01184	1.2621	0.2489
**Residual**	8.0949	0.47829		
**Total**	16.9248	1.00000		

Significance codes: **0.001 '***', 0.01 '**', 0.05 '*'**.

Note: R^2 indicates the proportion of total variance in microbial composition explained by each factor. The *F value* (pseudo-F) reflects the degree of compositional differences between groups defined by that factor; higher values suggest stronger group separation.

Table S5. Bacterial genera present in insectary and wild samples across analyzed conditions

Insectary

Paraherbaspirillum
Telluria

Wild

Abiotrophia
Achromobacter
Acidovorax
Actinomyces
Aeromicrobium
Altererythrobacter
Azotobacter
Blastocatella
Bosea
Bradyrhizobium
Brevundimonas
Campylobacter
Cedecea
Chryseobacterium
Citrobacter
Cloacibacterium
Desulfovibrio
Dialister
Dietzia
Elizabethkingia
Enterobacter
Fructilactobacillus
Gemella
Glutamicibacter
Gordonia
Haemophilus
Herbaspirillum
Hymenobacter
Jonquetella
Klebsiella
Kocuria
Lactobacillus
Lautropia
Leuconostoc
Marmoricola
Methylophilus
Methylopila
Microbacterium
Micrococcus
Moraxella
Negativicoccus
Neisseria
Ochrobactrum
Proteiniphilum
Proteus
Providencia
Pseudaminobacter
Rhodococcus
Solibacillus
Sphingopyxis
Tardiphaga
Variovorax
Weissella
Williamsia

Wild and Insectary

Acinetobacter
Aeromonas
Agathobacter
Aquitalea
Arsenophonus
Blastococcus
Brevibacterium
Budvicia
Comamonas
Corynebacterium
Cutibacterium
Delftia
Enhydrobacter
Enterococcus
Escherichia-Shigella
Flavobacterium
Hafnia-Obesumbacterium
Janthinobacterium
Knoellia
Lactococcus
Lawsonella
Lonsdalea
Mycobacterium
Nocardia
Pseudarthrobacter
Pseudomonas
Serratia
Shewanella
Sphingobium
Sphingomonas
Staphylococcus
Stenotrophomonas
Streptococcus
Tsukamurella
Yersinia

Insectary: Fasting

Knoellia
Shewanella

Insectary: Fed

Blastococcus
Escherichia-Shigella
Flavobacterium
Lonsdalea
Mycobacterium
Nocardia
Pseudarthrobacter
Sphingobium

Insectary: Fasting +TC

Agathobacter

Insectary: Fed +TC

Lactococcus

Insectary: Fasting and Fed

Brevibacterium
Corynebacterium
Cutibacterium
Enhydrobacter
Sphingomonas
Streptococcus
Tsukamurella

Insectary: Fasting, Fasting +TC and Fed

Lawsonella

Insectary: Fasting, Fed and Fed +TC

Telluria
Stenotrophomonas

Insectary all conditions

Acinetobacter
Aeromonas
Aquitalea
Arsenophonus
Budvicia
Comamonas
Delftia
Hafnia-Obesumbacterium
Janthinobacterium
Paraherbaspirillum
Pseudomonas
Serratia
Staphylococcus
Yersinia

Wild: Unfed

Altererythrobacter
Azotobacter
Lautropia
Pseudaminobacter

Wild: Unfed +TC

Budvicia

Wild: Fed +TC

Achromobacter
Acidovorax
Aeromicrobium
Bradyrhizobium
Citrobacter
Desulfovibrio
Fructilactobacillus
Hymenobacter
Marmoricola
Microbacterium
Moraxella
Ochrobactrum
Proteus
Sphingopyxis
Variovorax

Wild: Unfed and Fed +TC

Abiotrophia
Bosea
Campylobacter
Klebsiella
Methylophilus
Methylopila
Weissella

Wild: Fed and Fed +TC

Blastocatella
Blastococcus
Leuconostoc
Sphingobium

Wild: Unfed and Unfed +TC

Nocardia

Wild: Fed, Fed +TC and Unfed +TC

Brevibacterium
Knoellia
Providencia

Wild: Unfed, Fed and Unfed +TC

Cedecea
Lonsdalea

Wild: Unfed, Fed and Fed +TC

Chryseobacterium
Dialister
Jonquetella
Lactobacillus
Mycobacterium
Negativicoccus
Pseudarthrobacter
Proteiniphilum
Tardiphaga

Wild: Unfed, Fed +TC and Unfed +TC

Cloacibacterium
Elizabethkingia
Enterobacter
Enterococcus
Neisseria
Solibacillus

Wild all conditions

Acinetobacter
Actinomyces
Aeromonas
Agathobacter
Aquitalea
Arsenophonus
Brevundimonas
Comamonas
Corynebacterium
Cutibacterium
Delftia
Dietzia
Enhydrobacter
Escherichia-Shigella
Flavobacterium
Gemella
Glutamicibacter
Gordonia
Haemophilus
Hafnia-Obesumbacterium
Herbaspirillum
Janthinobacterium
Kocuria
Lactococcus
Lawsonella
Micrococcus
Pseudomonas
Rhodococcus
Serratia
Shewanella
Sphingomonas
Staphylococcus
Stenotrophomonas
Streptococcus
Tsukamurella
Williamsia
Yersinia

+TC: *T. cruzi* positive
Note: Genera highlighted green represents members of the core microbiota shared across all analyzed conditions.

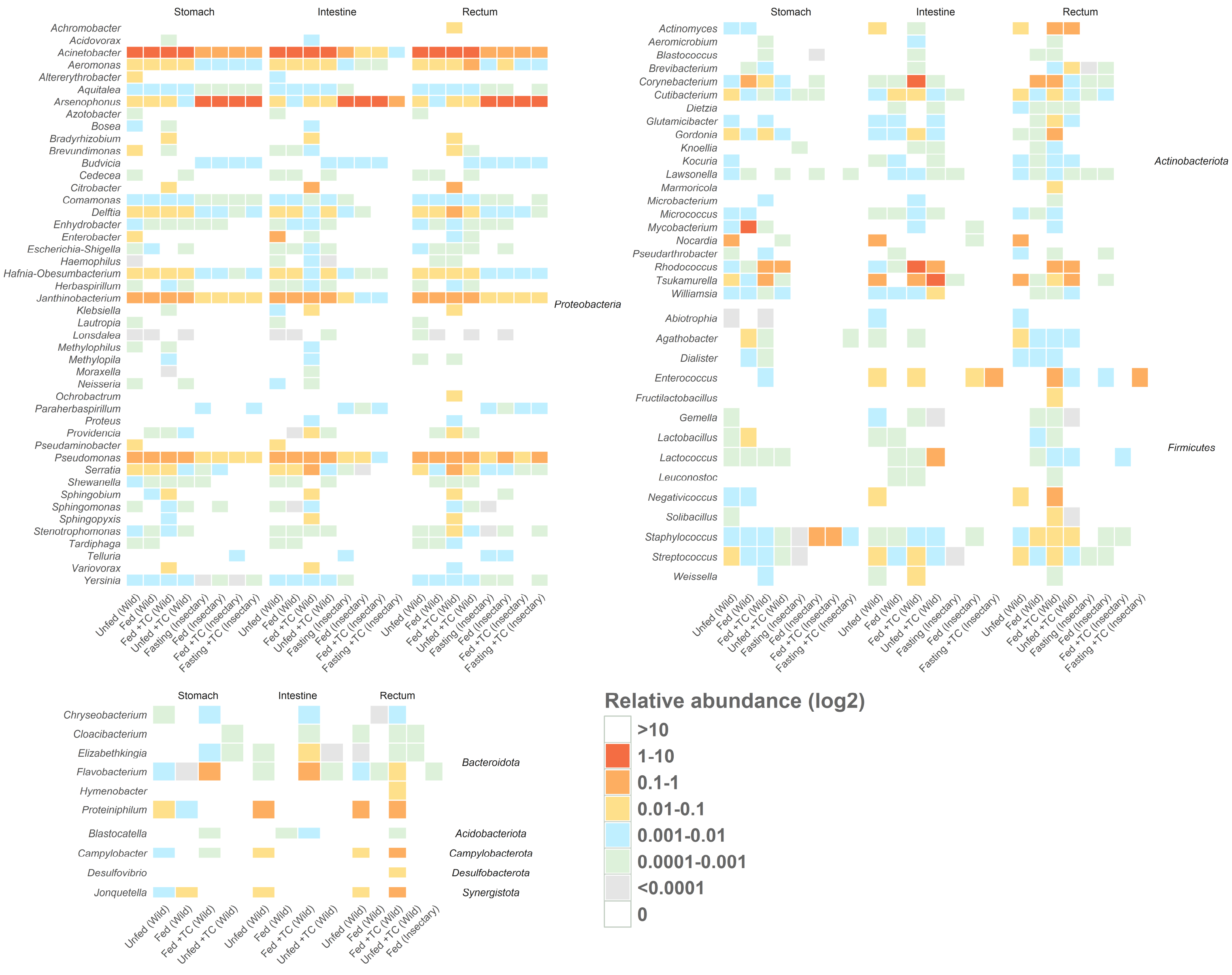

Figure S2. Log2-transformed the relative abundance of bacterial genera across gut regions in *T. pallidipennis* under insectary and wild conditions. Bacterial genera identified in the stomach, intestine, and rectum of *T. pallidipennis* were plotted by condition using $\log_2$-transformed relative abundance data derived from normalised 16S rRNA gene. Conditions included: Fasting (Insectary), Fed (Insectary), Fed +TC (Insectary), Fasting +TC (Insectary), Unfed (Wild), Fed (Wild), Fed +TC (Wild), and Unfed +TC (Wild). The heatmaps were segmented by phylum to preserve phylogenetic structure. Colour gradients represent relative abundance ranges, from high ($\log_2$ >10, red) to low (<0.0001, grey) and white absence. Genera such as *Pseudomonas* and *Acinetobacter* were widely distributed across organs from both origins but more consistently abundant in wild insects. Certain genera (e.g., *Arsenophonus* and *Janthinobacterium*) showed organ-specific patterns predominantly in insectary insects. Overall, the wild microbiota displayed broader genus diversity and higher abundance of values across organs.

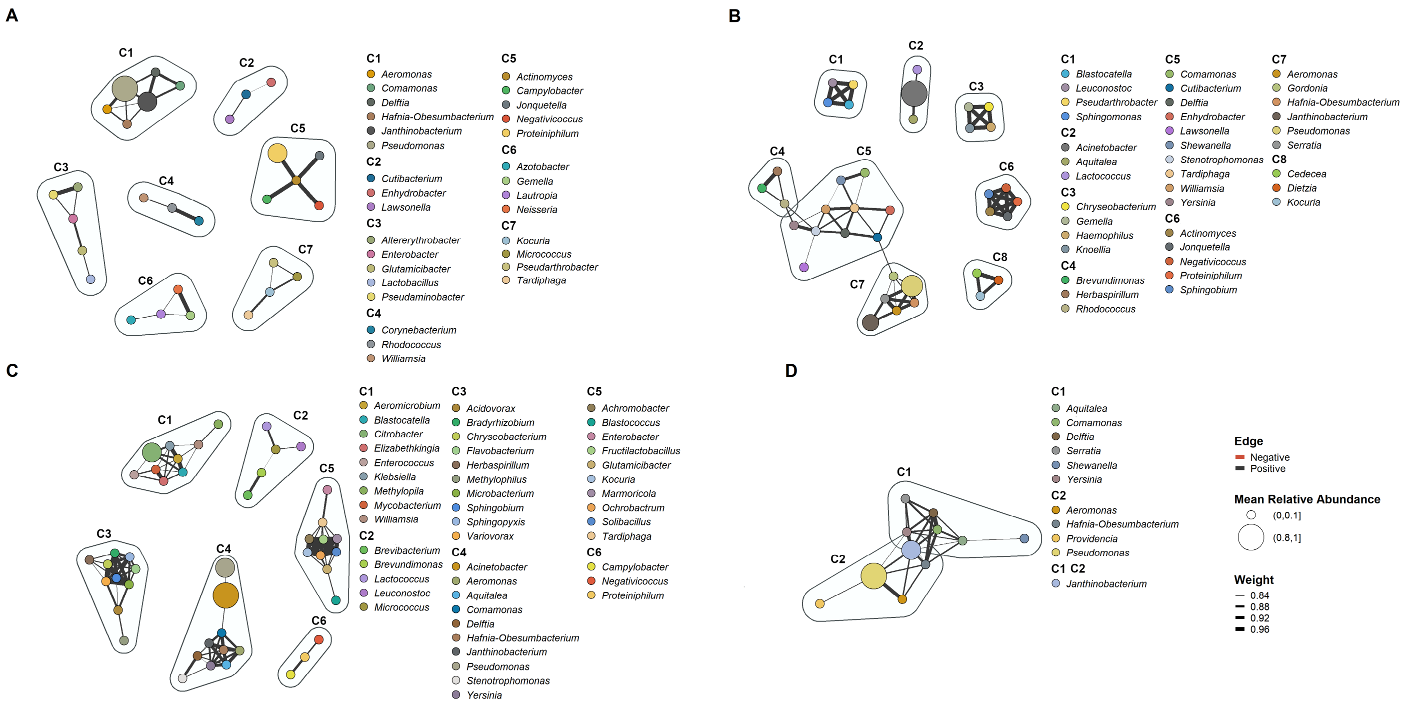

Figure S3. Co-occurrence network of the gut microbiota of *T. pallidipennis* under wild conditions. Networks were generated using the phylosmith package based on Spearman correlations (ρ ≥ 0.8, p ≤ 0.01) between bacterial genera. Panels represent wild insects in different conditions: (A) Unfed, (B) Fed, (C) Fed + *T. cruzi* (TC), and (D) Unfed +TC. Nodes indicate genera (size ∝ abundance), edges represent significant correlations (black: positive, red: negative), and edge thickness reflects correlation strength. In unfed wild insects, core genera like *Pseudomonas*, *Aeromonas*, and *Janthinobacterium* formed dense positive correlations (Cluster 1), while a separate cluster (C7) included Volatile Organic Compounds-associated (VOC-associated) taxa like *Kocuria* and *Micrococcus*. Feeding promoted additional clusters with genera such as *Sphingomonas*, *Leuconostoc*, and *Pseudarthrobacter* (C1). In the fed +TC condition, highly cohesive clusters emerged (e.g., *Ochrobactrum*, *Fructilactobacillus*, *Kocuria*. Under unfed +TC, core interactions persisted but with increased connections to genera like *Providencia* and *Serratia*. **See Table 1**. **+TC**: *T. cruzi* positive.

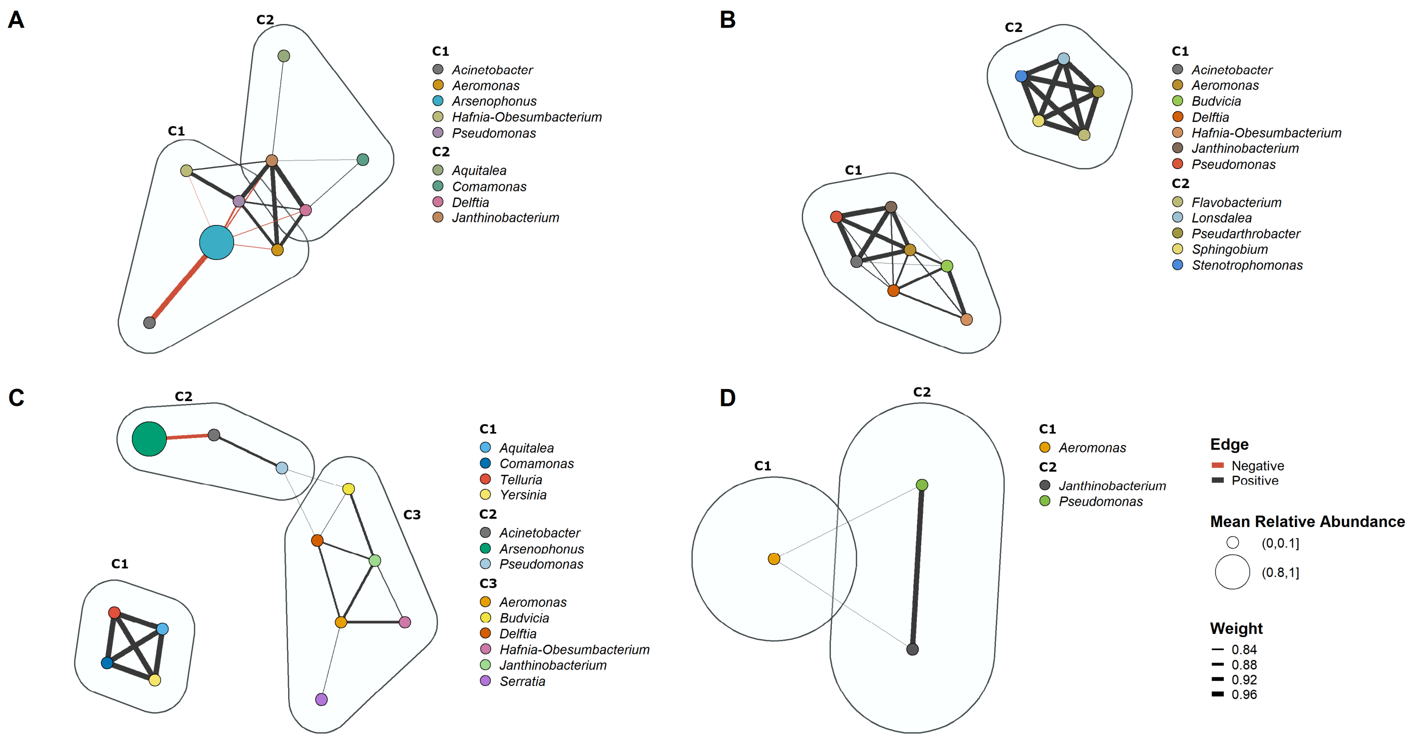

Figure S4. Co-occurrence network analysis of the gut microbiota of T. pallidipennis under insectary conditions. Networks were generated using the phylosmith package based on Spearman correlations ($\rho \geq 0.8$, $p \leq 0.01$) between bacterial genera. Panels represent insectary insects in different conditions: (A) Fasting, (B) Fed, (C) Fed + *T. cruzi* (TC), and (D) Fasting +TC. Nodes indicate genera (size ∝ abundance), edges represent significant correlations (black: positive, red: negative), and edge thickness reflects correlation strength. (A) Under fasting, *Arsenophonus* exhibited strong negative correlations with key genera such as *Acinetobacter*, *Pseudomonas*, and *Aeromonas*. (B) In the fed group, the network split into two tightly connected clusters: one comprising metabolically versatile genera like *Acinetobacter*, *Pseudomonas*, and *Aeromonas*, and another including *Flavobacterium*, *Stenotrophomonas*, and *Pseudarthrobacter*, indicating niche differentiation and possible synergistic functions. (C) In fed +TC insects, *Arsenophonus* maintained negative associations with core microbiota members, while genera involved in nutrient metabolism (*Aeromonas*, *Delftia*, *Janthinobacterium*) remained strongly co-associated. (D) In fasting + TC individuals, the network was simpler but retained a stable module formed by *Pseudomonas*, *Janthinobacterium*, and *Aeromonas*, reflecting a resilient core microbiota despite nutritional and infectious stress. **See Table 1**. +TC: *T. cruzi* positive.

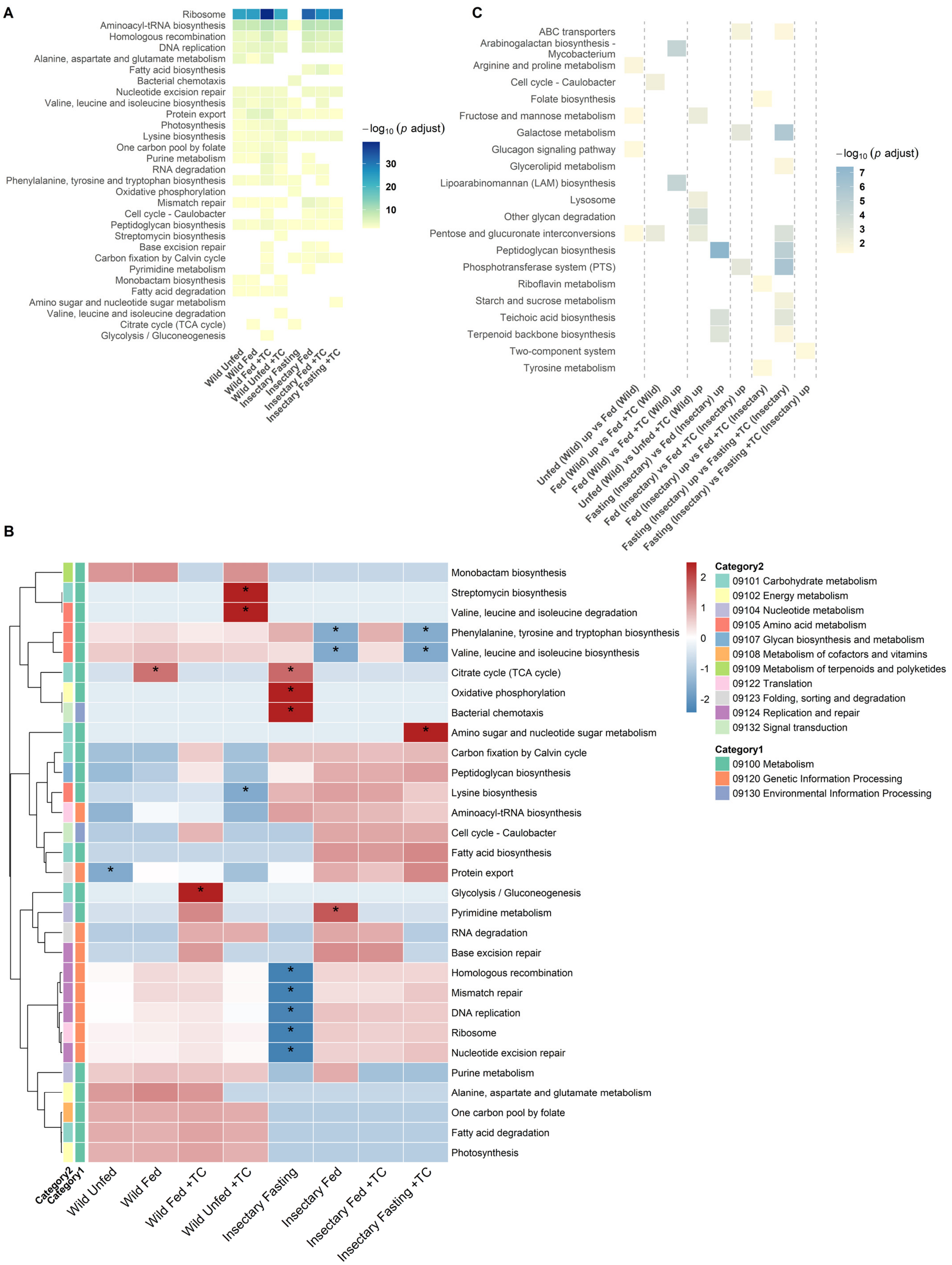
A
Ribosome
Aminoacyl-tRNA biosynthesis
Homologous recombination
DNA replication
Alanine, aspartate and glutamate metabolism
Fatty acid biosynthesis
Bacterial chemotaxis
Nucleotide excision repair
Valine, leucine and isoleucine biosynthesis
Protein export
Photosynthesis
Lysine biosynthesis
One carbon pool by folate
Purine metabolism
RNA degradation
Phenylalanine, tyrosine and tryptophan biosynthesis
Oxidative phosphorylation
Mismatch repair
Cell cycle - Caulobacter
Peptidoglycan biosynthesis
Streptomycin biosynthesis
Base excision repair
Carbon fixation by Calvin cycle
Pyrimidine metabolism
Monobactam biosynthesis
Fatty acid degradation
Amino sugar and nucleotide sugar metabolism
Valine, leucine and isoleucine degradation
Citrate cycle (TCA cycle)
Glycolysis / Gluconeogenesis
Wild Unfed
Wild Fed
Wild Fed +TC
Wild Unfed +TC
Insectary Fasting
Insectary Fed
Insectary Fed +TC
Insectary Fasting +TC
$-\log_{10}$ (p adjust)
30
20
10
C
ABC transporters
Arabinogalactan biosynthesis - Mycobacterium
Arginine and proline metabolism
Cell cycle - Caulobacter
Folate biosynthesis
Fructose and mannose metabolism
Galactose metabolism
Glucagon signaling pathway
Glycerolipid metabolism
Lipoarabinomannan (LAM) biosynthesis
Lysosome
Other glycan degradation
Pentose and glucuronate interconversions
Peptidoglycan biosynthesis
Phosphotransferase system (PTS)
Riboflavin metabolism
Starch and sucrose metabolism
Teichoic acid biosynthesis
Terpenoid backbone biosynthesis
Two-component system
Tyrosine metabolism
Unfed (Wild) up vs Fed (Wild)
Fed (Wild) up vs Fed +TC (Wild)
Fed (Wild) vs Fed +TC (Wild) up
Unfed (Wild) vs Unfed +TC (Wild) up
Fasting (Insectary) vs Fed (Insectary) up
Fed (Insectary) vs Fed +TC (Insectary) up
Fed (Insectary) up vs Fed +TC (Insectary)
Fasting (Insectary) up vs Fasting +TC (Insectary)
Fasting (Insectary) vs Fasting +TC (Insectary) up
$-\log_{10}$ (p adjust)
7
6
5
4
3
2
B
Monobactam biosynthesis
Streptomycin biosynthesis
Valine, leucine and isoleucine degradation
Phenylalanine, tyrosine and tryptophan biosynthesis
Valine, leucine and isoleucine biosynthesis
Citrate cycle (TCA cycle)
Oxidative phosphorylation
Bacterial chemotaxis
Amino sugar and nucleotide sugar metabolism
Carbon fixation by Calvin cycle
Peptidoglycan biosynthesis
Lysine biosynthesis
Aminoacyl-tRNA biosynthesis
Cell cycle - Caulobacter
Fatty acid biosynthesis
Protein export
Glycolysis / Gluconeogenesis
Pyrimidine metabolism
RNA degradation
Base excision repair
Homologous recombination
Mismatch repair
DNA replication
Ribosome
Nucleotide excision repair
Purine metabolism
Alanine, aspartate and glutamate metabolism
One carbon pool by folate
Fatty acid degradation
Photosynthesis
Category2
Category1
Wild Unfed
Wild Fed
Wild Fed +TC
Wild Unfed +TC
Insectary Fasting
Insectary Fed
Insectary Fed +TC
Insectary Fasting +TC
2
1
0
-1
-2
Category2
09101 Carbohydrate metabolism
09102 Energy metabolism
09104 Nucleotide metabolism
09105 Amino acid metabolism
09107 Glycan biosynthesis and metabolism
09108 Metabolism of cofactors and vitamins
09109 Metabolism of terpenoids and polyketides
09122 Translation
09123 Folding, sorting and degradation
09124 Replication and repair
09132 Signal transduction
Category1
09100 Metabolism
09120 Genetic Information Processing
09130 Environmental Information Processing

Figure S5. Functional enrichment of KEGG pathways across feeding conditions, *T. cruzi* infection, and environmental origin in *T. pallidipennis*. Functional enrichment analysis of predicted KEGG pathways was performed using PICRUSt2 based on 16S rRNA data, applying over-representation analysis (ORA) to the top 10% most abundant KOs (A), (B) z-score transformation to visualise deviations in pathway abundance across conditions, and (C) DESeq2 to identify differentially abundant KOs between pairs of conditions followed by pathway enrichment. (A) shows condition-specific enrichment of core pathways such as amino acid biosynthesis, nucleotide metabolism, DNA repair and translation, with insectary insects displaying consistent enrichment of ribosomal and replication-related functions, while wild insects exhibit broader metabolic versatility including photosynthesis and carbohydrate degradation. (B) reveals divergent z-score profiles, with insectary fasting samples enriched in oxidative phosphorylation and Tricarboxylic Acid cycle (TCA cycle), whereas wild feeding and infection promote glycolysis, antibiotic biosynthesis, and amino acid turnover. (C) confirms these patterns through differential analysis, highlighting increased replication and repair under feeding and infection, particularly in insectary groups, and greater metabolic reprogramming in wild insects exposed to *T. cruzi*. Collectively, the panels illustrate functional plasticity in the microbiota of *T. pallidipennis*, shaped by feeding state, parasite infection and environmental origin.
